# Matrix Metalloproteinase-sensitive Multistage Nanogels Promote Drug Transport in 3D Tumor Model

**DOI:** 10.7150/thno.34851

**Published:** 2020-01-01

**Authors:** Gregor Nagel, Ana Sousa-Herves, Stefanie Wedepohl, Marcelo Calderón

**Affiliations:** 1 Freie Universität Berlin, Institute of Chemistry and Biochemistry, Takustr. 3, 14195 Berlin, Germany.; 2 POLYMAT and Applied Chemistry Department, Faculty of Chemistry, University of the Basque Country UPV/EHU, Paseo Manuel de Lardizabal 3, 20018 Donostia-San Sebastián, Spain; 3 IKERBASQUE, Basque Foundation for Science, 48013 Bilbao, Spain.

**Keywords:** Multistage, fluorogenic, nanogels, matrix metalloproteinases, tumor spheroids

## Abstract

Physiological barriers inside of tumor tissue often result in poor interstitial penetration and heterogeneous intratumoral distribution of nanoparticle-based drug delivery systems (DDS). Novel, matrix metalloproteinase (MMP)-sensitive peptide-crosslinked nanogels (pNGs) as multistage DDS are reported with a beneficial size reduction property to promote the process of deep tissue penetration.

**Methods**: The presented pNGs are based on a dendritic polyglycerol (dPG) scaffold crosslinked by a modified MMP-sensitive fluorogenic peptide. The crosslinker integrates degradability in response to proteases present in the tumor microenvironment. Surfactant-free, inverse nanoprecipitation is employed to prepare the nanogels using strain-promoted click chemistry. The size and crosslinking density of the pNGs are controlled by the functionalization degree of dPG with cyclooctyne groups and by the peptide crosslinker fraction. The intrinsic reporter moiety of the crosslinker was used to study the influence of pNG compositions on the degradation profile. The therapeutic drug Doxorubicin was conjugated through a pH-sensitive linkage to dPG to form a multistage DDS. The penetration behavior of the pNGs was studied using agarose matrix and multicellular tumor spheroids (MCTS).

**Results**: Nanogel sizes were controlled in the range of 150-650 nm with narrow size distributions and varying degrees of crosslinking. The pNGs showed stability in PBS and cell media but were readily degraded in the presence of MMP-7. The crosslinking density influenced the degradation kinetic mediated by MMP-7 or cells. Stable conjugation of DOX at physiological pH and controlled drug release at acidic pH were observed. The digestions of nanogels lead to a size reduction to polymer-drug fragments which efficiently penetrated into agarose gels. Moreover, the degradable multistage pNGs demonstrated deeper penetration into MCTS as compared to their non-degradable counterparts. Thus, degradable pNGs were able to deliver their cargo and efficiently reduce the cell viability in MCTS.

**Conclusion**: The triggered size reduction of the pNGs by enzymatic degradation can facilitate the infiltration of the nanocarrier into dense tissue, and thereby promote the delivery of its cargo.

## Introduction

Despite all the advances that have been achieved in the field of nanocarrier-driven drug delivery, many challenges remain 1-5. One major obstacle is poor tumor penetration and heterogeneous distribution throughout the diseased tissue after extravasation of the nanoparticles from the vasculature 6, 7. While the accumulation of nanocarrier-based drug delivery in tumor tissue mediated by the enhanced penetration and retention (EPR) effect has been well established for many setups *in vivo*, the corresponding improvement of the therapeutic efficiency has often been deficient 7-9. This can be accounted, for instance, to the hampered penetration and uneven distribution of the nanocarrier in deeper regions of the tumor which result from heterogeneous vasculature, high interstitial fluid pressure, and dense interstitial matrix 7, 10, 11. Nanocarriers aiming for EPR-based delivery are typically in the size of 100-300 nm 6. However, it was found that particles in this size range cannot easily penetrate into the interstitial space 12, leading to a heterogeneous distribution of particles within the malignant tissue. This impairs the treatment, and additionally the barely perfused center of the tumor harbors the most aggressive cells that have the potential to regenerate the tumor if not eliminated completely 13, 14. Low exposure of drug in some regions of the tumor could even promote the development of drug resistance 15, 16. Conventional small molecule therapeutic agents show fast diffusion in tumor stroma, they are however also rapidly cleared from body and tissues.

One approach to improve intratumoral delivery is to carefully design multistage delivery systems that utilize specific stimuli in the tumor microenvironment such as pH or proteases to either achieve deeper tumor penetration, increased cellular uptake, and/or controlled drug release 17, 18. The nanocarriers possess design features that allow the response to specific stimuli encountered in a consecutive manner 19. Examples have been reported that respond to the slightly acidic tumor microenvironment (pH 6-7) with a size reduction by disintegration, sequential swelling/shrinking, or reorganization to enhance the diffusive properties of the particles 17, 20-22. However, the acidic pH is typically located far from the blood vessels (pH gradient) which would hinder the response in the perivascular regions. As enzymatic reactions are highly specific and enzyme activity is strongly dependent on location, cell type, or the state of the targeted tissue, introduction of enzyme-responsive moieties into nanocarriers can aid to adapt the responsiveness of a drug delivery system (DDS) to the abundant proteases in the tumor microenvironment 23, 24. Therefore, nanoparticles based on the natural polymers gelatin have been presented that encapsulate smaller entities like quantum dots or gold nanoparticles 25-27. The idea of these carriers is that the gelatin shell can be degraded by extracellular proteases and thereby release the smaller entities, which can then more readily diffuse within the dense interstitial space. Gelatin is a substrate for matrix metalloproteinases (MMPs), a family of extracellular endopeptidases with the ability to degrade components of the extracellular matrix (ECM). Upregulated MMP expression has been reported for several malignant conditions where they are involved in tumor proliferation and invasion as well as metastasis 28-32. The enzymes are located in the extracellular space and can be used for early detection and as biomarkers of disease progression and metastasis 33-35. Alternative MMP-responsive moieties are peptides that can be applied as cleavable linkages to introduce site-specific biodegradation or drug release 36-39.

Taking this into account, we designed multistage, MMP-sensitive, peptide-crosslinked nanogels (pNGs) that present suitable sizes to potentially accumulate in tumor tissue by the EPR effect and feature a protease-mediated size reduction property and acid-mediated drug release for enhanced tissue penetration. These degradable nanocarriers consist of a hydrophilic scaffold based on dendritic polyglycerol (dPG, Figure S1) that forms a three-dimensional network connected via MMP-specific peptide crosslinkers. The second building block is a rationally designed fluorogenic peptide crosslinker that comprises an MMP-specific amino acid sequence that is framed by a dye pair that exhibits fluorescence resonance energy transfer (FRET) and enables the monitoring of the peptide cleavage.^40^ The incorporation of the fluorogenic peptide as crosslinker integrates degradability to the nanocarrier comprised of otherwise non-degradable polymers, and additionally, the cleavage of the crosslinks can be conveniently monitored by the intrinsic fluorescence reporter. The design was inspired by hydrogels prepared by Janda and co-workers that include MMP-sensitive crosslinkers in acrylamide gels to introduce degradability into synthetic hydrogels 41. To transfer this concept to the nanoscale, we choose NGs as the nanometric equivalents of hydrogels that have emerged as platform for the development of novel nanocarrier-based strategies 42. NGs are soft, hydrophilic hydrogel particles formed by physically or chemically crosslinked polymer chains that can incorporate stimuli-responsive moieties into their network to enable a triggered reaction ranging from morphological changes such as swelling or shrinkage to the disintegration of the polymer network 43.

**Figure 1:**
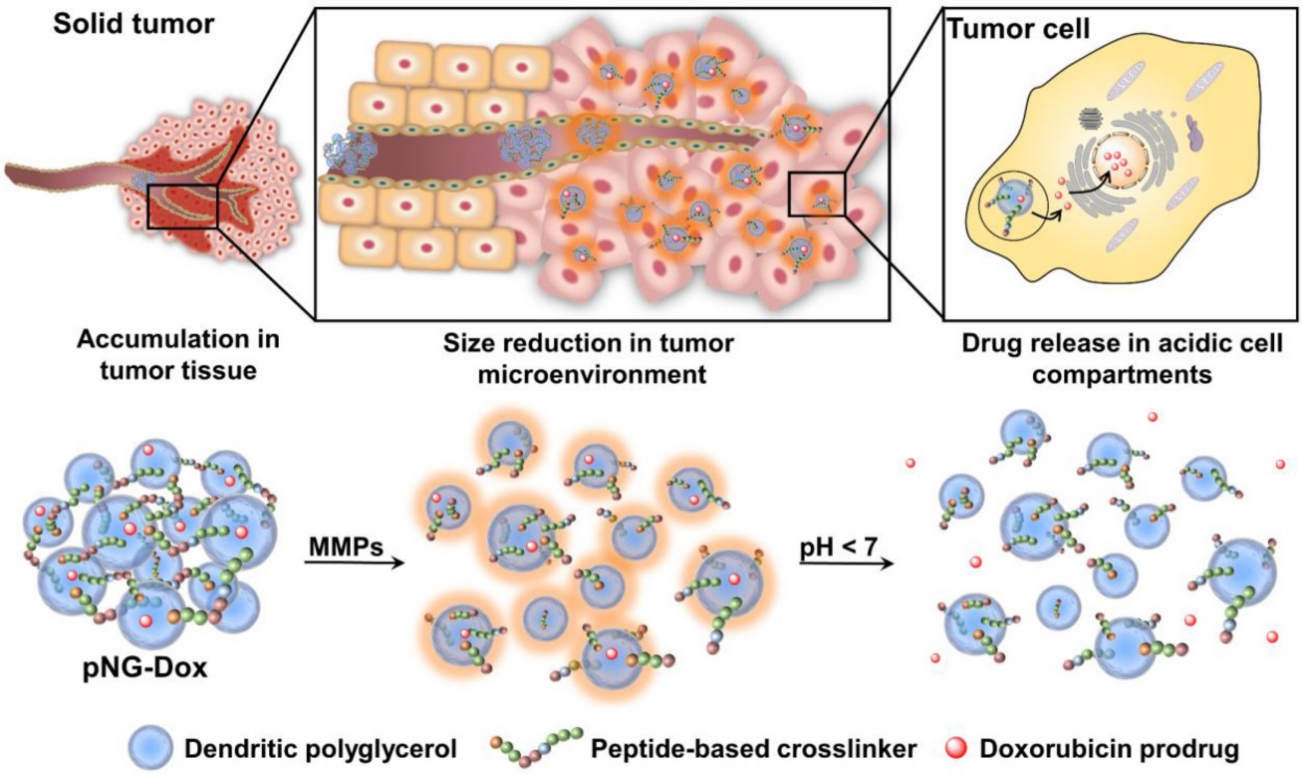
Schematic representation of the mechanism proposed for the multistage drug delivery by pNG-DOX.

Classically, NGs are prone to efficiently encapsulate their cargo by physical interactions in the network structure. However, encapsulation can cause premature release of the payload by diffusion mediated leakage before reaching the site of action. Additionally, the release of the drug should be prevented after MMP-mediated degradation as this could cause fast clearance of the small molecular weight drugs. As an alternative, covalently bound therapeutics can prevent leakage, mediate enhanced solubility, and, when attached by dynamic covalent chemistry, can be released in a controlled manner 44. To this end, we aimed to conjugate the chemotherapeutic drug doxorubicin (DOX) through a pH-sensitive linker to the dPG scaffold, thereby creating a multistage delivery system (pNG-DOX). In the first stage, pNGs can be degraded by MMPs which are present in the tumor microenvironment. After the resulting size reduction, the fragments constitute polymer-drug conjugates consisting of polyglycerol as polymeric carrier and DOX conjugated through an acid-cleavable linkage. These fragments should facilitate the penetration into deeper areas of tumor tissue where the cargo could be released at acidic pH as present in intracellular compartments (pH 4-5) such as endosomes and lysosomes (Figure 1) 45.

We expect that the synthetic approach based on multifunctional building blocks and the directed incorporation of cleavable peptides allows precise control over the size and crosslinking density of the pNGs by varying the applied feed. Different crosslinking densities should result in different degradation profiles which can be tuned towards the specific application as multistage nanocarrier. In our system, the MMP-induced size reduction and degradation can be conveniently studied using the intrinsic fluorescence probe of the crosslinker. To prove the principle of the multistage drug delivery system, we investigate the diffusive transport of MMP-digested pNGs in a dense gel matrix mimicking the ECM. Furthermore, we expect an enhanced penetration of the carrier fragments and the drug into multicellular tumor spheroids (MCTS) which should result in an improved therapeutic activity of the drug.

So far, the majority of the peptide-crosslinked nanocarriers only described the degradation to small fragments to release encapsulated cargo. To the best of our knowledge, the controlled introduction of peptide-based crosslinks into nanogels and the application as multistage delivery system has not been reported so far.

## Methods

### General Methods

All chemicals were purchased from Acros Organics, Alfa Aesar, Roth, Merck, Sigma-Aldrich (now Merck), Deutero GmbH, and used as received. The design of the peptide crosslinker was adapted and modified from the work of Janda et al. 41 and the strategy of the crosslinker synthesis was developed in cooperation with Protein Research Inc. (UK). Structure and peptide sequence: Mca-Lys((OEG)8-N3)-Pro-Leu-Gly-Leu-Lys(Dnp)-Ala-Arg-Lys((OEG)8-N3)-NH2 (Mca: 7-methoxycoumarinyl-4-acetic acid; Dnp: 2,4-dinitrophenyl; EG: ethylene glycol). Dendritic polyglycerol (dPG) was purchased from Nanopartica GmbH (Germany) with a weight average molecular weight (M*_w_*) of 10 kDa (Dispersity Đ = 1.27). The cyclooctyne reagent (1R,8S,9s)-Bicyclo[6.1.0]non-4-yn-9-ylmethyl N-succinimidyl carbonate was purchased from Synaffix (AE Oss, Netherlands). Water used for the synthesis was obtained from a Millipore water purification system. The pH-sensitive (6-maleimidocaproyl) hydrazone derivative of DOX (aldoxorubicin), was synthesized starting from 6-aminocaproic acid following a procedure from literature 46. ^1^H NMR and ESI-MS spectra are shown in the SI.

HeLa cells (DSMZ-No. ACC-57, Leibnitz Institute DSMZ- German Collection of Microorganisms and Cell Cultures) were routinely maintained in RPMI 1640 medium (Lonza) containing 10% fetal bovine serum (FBS Superior, Merck), 1% Penicillin/Streptomycin (P/S, Thermo Fisher Scientific), and 1% MEM non-essential amino acids (Sigma-Aldrich) at 37 °C and 5% CO_2_. Human dermal fibroblasts from juvenile foreskin were isolated in accordance to local ethics and biosafety regulations (ethical approval EA1/345/14 by the Charité ethical committee) and were routinely cultured in Dulbecco's Modified Eagle Medium (DMEM) with 15% FBS and 1% P/S at 37 °C and 5% CO_2_.

### Functionalization of dPG

The hydroxyl groups of dPG were modified in three steps to obtain amine functionalities following published procedure (Scheme S1) 47. Briefly, dried dPG (250 mg dPG, 0.27 mmol OH's for 8%, 1.0 equiv.) was dissolved in *N,N*-dimethylformamide (DMF; 25 mL) with triethyl amine (113 µL, 0.81 mmol, 3 equiv.) and cooled down to 0 °C. The hydroxy groups were activated by addition of methanesulfonyl chloride (23.0 µL, 0.30 mmol, 1.1 equiv.). The reaction was stirred overnight (18 h) and allowed to reach rt. The reaction mixture was diluted with MeOH (4:1 v/v) and dialyzed against MeOH for 2 d (Molecular weight cut-off (MWCO) 1000 Da). The degree of functionalization was determined by ^1^H NMR. Degrees of functionalization for dPG are given as a percentage of the total dPG hydroxyl groups (~ 135 hydroxyl groups for 10 kDa dPG). For the next step, the polymer was dissolved in DMF and sodium azide (87.8 mg, 1.35 mmol, 5 equiv.) was added to induce a nucleophilic substitution of the mesyl groups. The reaction mixture was heated to 60 °C and stirred for 3 d. After filtration, the filtrate was diluted with MeOH, dialyzed against MeOH for 2 d (1000 Da MWCO) to obtain dPG-azide. The appearance of a characteristic azide band was observed in the IR spectrum (2100 cm^-1^) and the disappearance of the NMR signal for mesyl groups was monitored. To obtain dPG with amine functionalization, the azide groups were reduced by Staudinger reaction. Therefore, dPG-azide was dissolved in water/THF (1:1 v/v) and triphenylphosphine (213 mg, 0.81 mmol, 3 equiv.) was added before the solution was stirred for 3 d at 40 °C. Afterwards, TFA was evaporated and the remaining aqueous solution was filtered. The solution was dialyzed against MeOH for 3 d (MWCO 1000 Da). By this method dPG-amine with 4% and 8% degree of functionalization were obtained as determined by ^1^H NMR. dPG-amine war stored as solution in MeOH to avoid crosslinking and the concentration was determined gravimetrically after drying an aliquot of the solution.

### Functionalization of dPG with bicyclononyne groups (BCN)

For the functionalization with cyclooctyne groups, dPG-amine (250 mg, 0.27 mmol amine groups for 8%, 1.0 equiv.) was dissolved in DMF (20 mL) before triethylamine (112.3 µL, 0.81 mmol, 3.0 equiv.) and (1R,8S,9s)-Bicyclo[6.1.0]non-4-yn-9-ylmethyl N-succinimidyl carbonate (86.5 mg, 0.30 mmol, 1.1 equiv.) were added (Scheme S2). The reaction was stirred for 3 h at rt. Then, the solution was diluted with MeOH (4:1 v/v) and dialyzed against MeOH for 3 d (MWCO 1000 Da). Afterwards, the dialysis medium was changed to water for 2 d. dPG-BCN was stored in aqueous solution and the concentration was determined gravimetrically after an aliquot of the solution was lyophilized. The degree of functionalization was determined by ^1^H NMR yielding dPG-BCN with 3.9% and 7.8% conversion.^ 1^H-NMR (500 MHz, D_2_O, δ: 4.24-3.42 (m, 5H, dPG backbone), 2.31-2.14 (m, 6H, cyclooctyne), 1.60-1.28 (m, 2H, cyclooctyne), 0.98-0.80 (m, 3H, cyclopropane) ppm.

### Preparation of pNGs

For the synthesis of the pNGs, nanoprecipitation was employed using different degrees of BCN-functionalization and different feed ratios (see Table S1). As an example, dPG-BCN (4% functionalization, 4.1 mg, 2.2
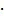
10^-3^ mmol BCN groups) and peptide crosslinker (2.0 mg, 1.6
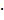
10^‑3^ mmol azide groups, 70 mol%) were dissolved separately in water (1 mL) and cooled down in an ice bath. The cooled solutions were mixed and directly injected into NaCl-saturated acetone (20 mL) under vigorous stirring (900 rpm). After injection, the stirring was stopped, and the nanoprecipitation was left for 2 d at rt before the excess of BCN groups was quenched with either azidopropanol, indocarbocyanine azide (ICC-azide) or 11-Azido-3,6,9-trioxaundecan-1-amine to obtain pNG-OH, pNG-ICC or pNG-NH_2_, respectively. The dispersion was left for 1 d more before of water (1 mL) was added and acetone was evaporated. The aqueous dispersion was dialyzed against water for 3 d (MWCO 50 kDa). For the preparation of non-degradable controls, the same methodology was applied using the peptide crosslinker comprised of d-amino acids. pNGs were characterized by DLS and UV/Vis spectroscopy.

### Synthesis of multistage pNGs (pNG-DOX)

pNGs (5 mg) quenched with 11-Azido-3,6,9-trioxaundecan-1-amine were reacted with 2‑iminothiolane hydrochloride (1.1 mg, 8.1
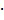
10^-3^ mmol, 3 equiv. of maximum amine groups) to convert the amine groups to thiols. After 20 min, the dispersion was filtered through a desalting column (PD10 column; GE Healthcare) to separate remaining 2‑iminothiolane. The pNGs dispersion was concentrated by centrifugal filter devices (Vivaspin ®, MWCO 100 kDA) before aldoxorubicin (2.1 mg, 2.7
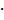
10^-3^ mmol, 1 equiv.), dissolved in DMF (0.1 mL), was added and left to stir for 4 h at rt. The pNGs were purified by size exclusion chromatography (SEC) using Sephadex G‑25 fine matrix and subsequently dialyzed for 2 d in water (MWCO 50 kDa). Size distribution and zeta potential of pNGs were measured at 25 °C by DLS. The loading of labeled indocarbocyanine azide (ICC) or conjugated DOX was determined by UV-Vis spectroscopy. The NGs were stored as highly concentrated dispersions (5 mg mL^-1^) at 4 °C. The same methodology was applied for the preparation of non-degradable controls using the peptide crosslinker comprised of d-amino acids.

### Degradation study of pNG

#### By dynamic light scattering (DLS)

To follow the degradation of the pNGs by DLS, recombinant human MMP-7 (R&D Systems) were activated at 100 µg mL^-1^ with 1 mM p-aminophenylmercuric acetate (APMA) in a solution of 50 mM Tris base, 10 mM CaCl_2_, 150 mM NaCl, 0.05% (w/v) Brij-35, and pH 7.5 (TCNB) for 1 h at 37 °C. The enzyme solution was diluted to 0.4 µg mL^-1^. 50 µL of NGs solution at 1 mg mL^-1^ were filled into low volume cuvettes (Sarstedt) and placed in the Zetasizer Nano-ZS 90 (Malvern). The reaction was initiated by addition of enzyme solution (50 µL at 0.4 µg mL^-1^). The particle size distributions were measured every 30 min at 37°C over 16 h.

#### By fluorescence

The degradation was monitored by fluorescence intensity measurements over time following the fluorescence of 7-methoxycoumarin. pNG solutions of 0.01-1.0 mg mL^-1^ were prepared in TCNB buffer and 50 µL were loaded into a 96-well microplate. The reaction was started by adding 50 µL of the activated MMP-7 solution (0.02 µg per well). Plates included substrate and background controls containing pNGs with buffer and buffer only. As a positive control, a fluorogenic peptide (Mca-Pro-Leu-Gly-Leu-Dpa-Ala-Arg-NH_2_, R&D systems) was included at a concentration of 10 µM to confirm enzyme activity. The microplate was sealed with optically clear adhesive seal sheets (Absolute qPCR Seal, Thermo Scientific) and placed into a microplate reader (Infinite M200 Pro, Tecan) heated to 37 °C. The excitation and emission wavelengths were set to 320 nm and 405 nm (top read), respectively, and fluorescence intensity was recorded every 5 min for 15 h. The background signals of pNGs in assay buffer were subtracted and the fluorescence intensities, expressed as changes relative to the starting point, were plotted versus time.

### Release of DOX

pNG-DOX dispersions in H_2_O were mixed with different buffers (1:1 v/v with acetate buffer (50 mM sodium acetate/acetic acid, 150 mM NaCl) at pH 5 or Tris buffer (50 mM Tris base, 10 mM CaCl_2_, 150 mM NaCl, 0.05% w/v Brij-35) at pH 7.5 with or without MMP-7) at 5 mg mL^-1^ and incubated at 37 °C. At specific time points (t = 0 min, 1 h, 2.5 h, 5 h, 8 h, 24 h) an aliquot of the solution was transferred to a SEC column containing Sephadex G-25 fine matrix to separate the free DOX from the NGs. The collected NG fraction was analyzed for remaining DOX by UV/Vis spectroscopy (absorption at 490 nm).

### Diffusion in agarose gels

Agarose gels were prepared by heating a suspension of agarose in phosphate buffered saline (PBS; 0.5 w%) in a microwave oven at 500 W for 30 seconds. The clear agarose solution was filled into rectangular capillaries of borosilicate glass (Hilgenberg, LxBxW: 80x4.2x1.25 mm, wall thickness 120 µm). After gel formation at rt, NGs labeled with ICC (pNG-ICC, 0.1 mg) were incubated with either MMP-7 (final concentration 0.02 µg mL^-1^) in TCNB buffer or in buffer alone. After 16 h, ethylenediaminetetraacetic acid (EDTA) was added to inactivate the enzyme and 20 µL of the solution was filled into the capillaries on top of the agarose gel. In addition, free ICC in the same concentration was added to a capillary and then, the capillaries were kept at 37 °C in a humidified chamber for another 16 h. The gels were imaged using a gel imaging system (GelDoc XRS+, Bio Rad) with green epi illumination (0.2 s exposure time) and 605/50 nm filter. The images were analyzed by ImageJ software.

### MCTS penetration

After 9 d in culture (described in SI), spheroids were rinsed twice with PBS before fluorescently labeled pNGs-ICC, degradable and non-degradable control, or free ICC in RPMI medium without FBS and phenol red were added to the spheroids. The pNGs were added to a final concentration of 1.5 µM regarding the fluorescent dye. The spheroids were incubated for 2 or 16 h at 37 °C and 5% CO_2_. Afterwards, the medium was discarded, spheroids were washed three times with PBS, and fixed for 40 min at rt in 10% neutral buffered formalin. Spheroids were washed again twice with PBS and cell nuclei were stained with DAPI solution (2.5 µg mL^-1^ in PBS, Sigma) for 1 h at rt. After washing with PBS, two complementary methods were applied to realize the visualization of the pNGs penetration into MCTS. For the first one, spheroids were transferred using 1 mL pipetting tips onto microscope slides, squeezed under microscopy cover slips and mounted with ProTaqs MountFluor (Quartett GmbH) mounting medium. For the second method, the fixed spheroids were stained with methylene blue (0.1% in PBS) for 10 min at rt. After washing with PBS, spheroids were transferred to Peel-A-Way® S-22 embedding molds (Merck) embedding molds and embedded in Surgipath FSC 22 clear embedding compound (Leica) before the samples were frozen using liquid nitrogen. Then, the spheroids were cut into 16 µm-thick sections and mounted onto microscope slides. Images were acquired with a Leica SP8 CLSM using laser excitation at 488 nm (DOX) and 561 nm (ICC) with 20-fold and 64-fold magnification. Image analysis was performed with LASX software and ImageJ. The circumference was determined in the brightfield images and then transferred to the intensity images. Subsequently, the measuring functions was used to determine the mean fluorescence intensity in the area of the MCTS. The same procedure was followed for analyzing the penetration of multistage pNGs (degradable and non-degradable and free DOX) into spheroids. Here, a concentration of 5µM regarding DOX was applied for all samples.

## Results and Discussion

We propose the use of peptide crosslinked nanogels (pNGs) as a multistage drug delivery system (DDS) that features MMP-mediated size reduction properties in response to the tumor microenvironment followed by controlled drug release at intracellular, acidic pH conditions to aid enhancing tumor tissue penetration of conjugated DOX. To design a suitable pNG for this purpose, we considered the initial size of the pNG that potentially enables accumulation in tumor tissue by the EPR effect. This requires rather large particle sizes of 100-300 nm 9. In the tumor environment, extracellular MMPs would cleave the peptide linkers and thereby, cause a size reduction by degradation of the pNG to smaller fragments which should facilitate deeper tissue penetration. The size of the degradation products must be carefully controlled, since particles smaller than 30 nm may be cleared from tumor tissue while larger particles are retained and continue to accumulate 48. It has been reported that micelles with sizes around 50 nm are able to penetrate tumor tissues and at the same time are more likely to be retained in the tumor tissue while smaller micelles of 30 nm are susceptible to more rapid clearance 6, 8, 12. Therefore, we first systematically investigated the synthetic parameters and the influence of the different building blocks on the resulting properties of the pNGs with the goal to find a suitable candidate for the proposed multistage DDS.

### Formation and characterization of pNGs

**Figure 2:**
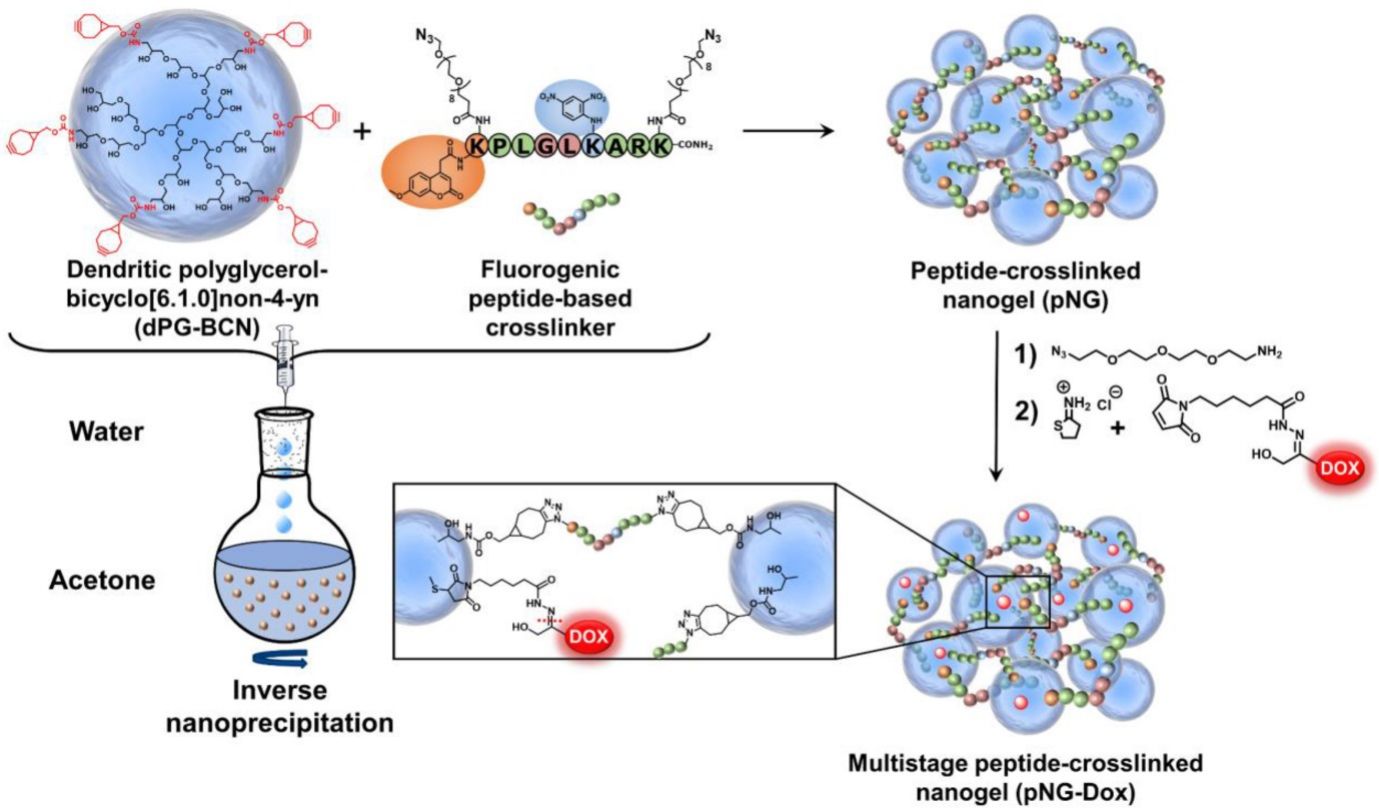
Schematic representation of the building blocks, synthesis, and structure of multistage pNG-Dox.

In the first step, we developed the methodology for the preparation of the MMP-sensitive NGs. The pNGs consist of dPG crosslinked by a fluorogenic peptide that can be cleaved by MMPs, as well as a chemotherapeutic drug attached via an acid labile linker to the polymer. The hyperbranched polymer dPG was chosen to form the hydrophilic scaffold of the pNGs and to allow facile functionalization 49. Furthermore, dPG served as polymeric carrier of DOX after fragmentation. The hydroxy groups of dPG were converted to amine groups in a three-step approach following a known procedure 47. Afterwards, the amines were reacted with (1R,8S,9s)-Bicyclo[6.1.0]non-4-yn-9-ylmethyl N-succinimidyl carbonate to introduce cyclooctyne groups (dPG-BCN, Figure 2).

To employ the fluorogenic peptide as crosslinker, it was modified with two terminal azide groups. These groups were attached through the γ-amino groups of lysine side chains including an 8-unit oligoethylene glycol (OEG)-chain as spacer leading to the final structure: Mca-Lys((OEG)8-N3)-Pro-Leu-Gly-Leu-Lys(Dnp)-Ala-Arg-Lys((OEG)8-N3)-NH2 (Figure 2). The additional OEG chains were introduced to increase water solubility and accessibility for the proteases. The functional groups allow strain-promoted alkyne-azide cycloaddition (SPAAC), a biorthogonal, metal-free click reaction that can be performed under mild conditions. Therefore, the reaction type allows the crosslinking of the building blocks without damaging the sensitive peptide and potentially enables the encapsulation of sensitive cargos 50.

**Figure 3:**
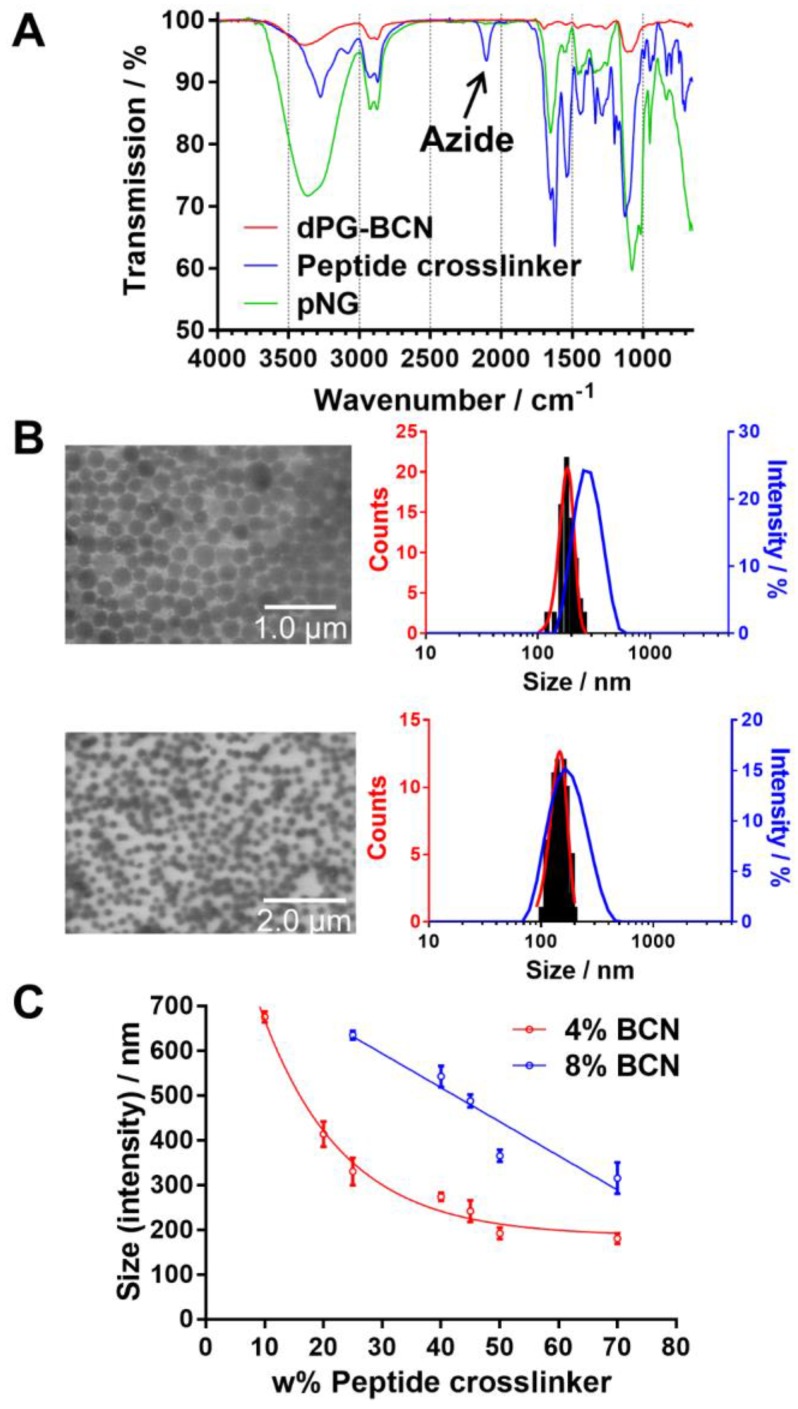
Characterization of pNGs. a) FT-IR spectra of dPG-BCN (red), peptide crosslinker (blue), and pNGs (green). b) Representative TEM images of pNGs with 4% BCN functionalization and 40 w% (pNG4) and 70 w% (pNG7) peptide crosslinker and the corresponding statistical analysis of the particle size distribution (red) and the DLS measurement (blue). c) Influence of the peptide crosslinker feed and dPG functionalization on pNGs sizes.

NGs are usually prepared by templating the polymer in confined spaces and subsequent crosslinking inside these templates 51. The most common methods are the mini- or microemlusion 52. However, the high shear stress impairs the encapsulation of sensitive cargos and substantial amount of surfactant may alter surface properties when not carefully removed during purification. To bring the presented building blocks into nanosized assemblies, we employed the inverse nanoprecipitation technique 53-55. This mild method avoids extended stirring/sonification (shear stress) and is surfactant-free. For the inverse nanoprecipitation, water was used as solvent and acetone was chosen as non-solvent. A constant polymer concentration of 3 mg/mL and a solvent/non-solvent ratio of 1:20 were selected as working parameters. These conditions were found to be optimal to obtain stable dispersions and were maintained for the synthetic screening. The cyclooctyne functionalities were used in excess and were quenched with azidopropanol, or alternatively, with an azide-functionalized indocarbocyanine dye (ICC) as florescent label. The crosslinking reaction was monitored by FT-IR spectroscopy which showed the disappearance of the azide signal at 2100 cm^-1^ indicating the formation of triazoles by SPAAC (Figure 3a).

We hypothesized that by varying the fraction of the peptide crosslinker in the feed and the crosslinking points on the dPG surface, different sizes and network densities would be obtained. The size is crucial for the fate of the pNGs in biological environment and the network has an influence on the degradation rate of the pNG which can be optimized for size reduction ability or alternatively to tune the release of an encapsulated cargo. Therefore, a rational screening included the variation of dPG-BCN functionalization degree and the fraction of peptide crosslinker in the feed to study their effect on the composition of the polymeric particles. Two degrees of BCN functionalization with 4% and 8% of converted hydroxy groups (~5 and 10 groups per dPG) were tested and the peptide crosslinker feed was varied from 10 to 70 w%. The hydrodynamic diameters and polydispersity indices (PDI) as determined by dynamic light scattering (DLS) showed narrow, monomodal distributions for the prepared pNGs (Table S1). To complement these results, we confirmed the formation of spherical particles by transmission electron microscopy (TEM) measurements. The sizes determined by statistical analysis of the TEM images revealed smaller diameters compared to the DLS measurements, which is due to drying and associated deswelling of the particles. For example, particle sizes of ~270 nm were determined for pNG4 by DLS, while TEM images revealed sizes of ~180 nm. Interestingly, pNG7 with a higher peptide feed displayed a size of 180 nm measured by DLS and only slightly smaller sizes of 150 nm were determined by TEM indicating a denser network (Figure 3b).

Overall, the screening of BCN functionalization and peptide feed confirmed that the sizes can be modulated in a broad range between 150 and 650 nm (Figure 3c). As expected, the nanogel size depends on the weight fraction of the peptide crosslinker. Here, smaller particle sizes were obtained with increasing peptide feed. The higher amount of crosslinker allows a stronger interconnection and additionally may provide stabilization during the nucleation and aggregation process yielding smaller particles. For higher BCN functionalization, we expected denser and smaller structures. Interestingly, an increase in sizes was observed when the number of BCN groups was doubled while maintaining the same peptide fraction. Besides the solvent/non-solvent and polymer concentration, eventually, the character of the polymer is responsible for the stabilization of the aggregates to prevent coalescence or Ostwald ripening in the nanoprecipitation process 56. Therefore, a higher functionalization of dPG seems to impair the potential of the polymer to stabilize smaller aggregates that has been observed for other dPG-based nanoparticles prepared by inverse nanoprecipitation 53, 55. Our screening revealed that the stabilization by dPG in inverse nanoprecipitation is reduced by functionalization with hydrophobic BCN groups. Nevertheless, the tested feed composition allows an application-optimized size adaptation in a broad size range. Additionally, hydrodynamic diameters for pNGs in different media including Milli-Q water, PBS, cell culture medium (RPMI), and serum-containing solution (10% human serum in PBS) were determined. Here, small size changes were observed for nanogels in saline media (PBS, TCNB buffer and RPMI; table S2). The sizes measured in water exhibit sizes that are 30% bigger than in saline-containing solutions indicating a higher swelling capability. It can be noted that no formation of larger aggregates was observed either in cell culture medium or in serum-containing solution.

### Size reduction and degradation kinetics of pNGs

**Figure 4:**
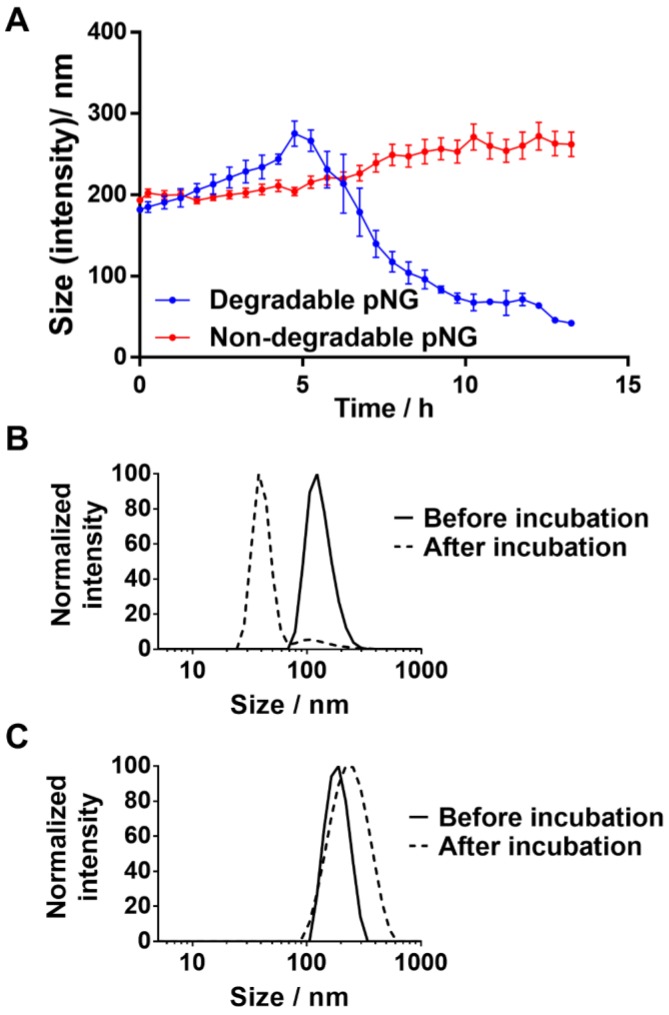
a) Hydrodynamic diameters of degradable or non-degradable pNGs after addition of MMP‑7 (at t=0) over time. Error bars indicate SEM from three measurements. Normalized size distributions of b) degradable and c) non-degradable pNG before and after incubation with MMP‑7 for 16 h.

For the MMP-specific peptide sequence, it has been reported that MMP-7 is able to efficiently hydrolyze the crosslinker sequence which resulted in an increased fluorescence signal when incubated with the fluorogenic crosslinker (Figure S2a) 41, 57. To confirm the degradability of the prepared nanogels, pNG6 (BCN 4%, 50 w%) was incubated in the presence and absence of the protease MMP‑7. Subsequently, particle sizes were monitored by DLS over time. In the first 4 hours, we observed a slight increase in size indicating a swelling of the particles, which we think is due to partial cleavage of crosslinking points, and hence loosening of the network. After this time, apparent particle sizes were decreasing suggesting a disintegration of the pNG networks to smaller portions (Figure 4a). For pNG6, size reduction of approximately 75% compared to the initial size was observed after 16 h (Figure 4b). As controls for the specific degradation, pNGs were prepared by the same procedure but using a peptide crosslinker comprised of D-amino acids, which is not hydrolyzed by MMP-7 (Figure S2b). As seen in Figure 4c, these non-degradable pNGs were stable upon incubation with the protease. These results confirm the MMP-mediated degradability and consequently a size reduction of the pNGs. The degradation was also confirmed by GPC measurements. Here, longer retention times for pNGs were observed after incubated with MMP-7 indicating fragmentation of the pNGs (Figure S3). It is well understood that MMPs play a pivotal role in the proliferation, invasion and metastasis of cancer since MMPs are one of the major class of enzymes to degrade the extracellular matrix, and thereby remodeling the tumor microenvironment 28. MMP expression is upregulated at all stages of cancer; however, the levels of MMPs vary significantly between different cancer types and patients. Many factors influence the activity of the proteases such as local concentrations, presence of inhibitors, and level of infiltrating stromal cells, which are the major source of MMPs. Isaacson et al. have thoroughly reviewed the MMP upregulation, distribution as well as their role in cancer 58. To illustrate the variation in MMP expression, the cancer tissue concentration of MMP-7 in colorectal cancer was determined to be 2.45 ng mg^-1^ whereas in pancreatic cancer was 143 ng mg^-1^. For all cancer types, an average value for MMP-7 of 20.9 ng mg^-1^ tissue was given. To induce the degradation of the pNGs, we employed an activated MMP-7 concentration of 200 ng ml^-1^ (≡ 0.2 ng mg^-1^; d = 1.0 kg m^-3^) in all assays. This corresponds to an MMP concentration which is one magnitude smaller compared to the conditions in colorectal cancer. Therefore, the applied MMP concentration is representative for conditions where MMP upregulation is moderate, but still higher than in healthy tissue and in the bloodstream.

**Figure 5:**
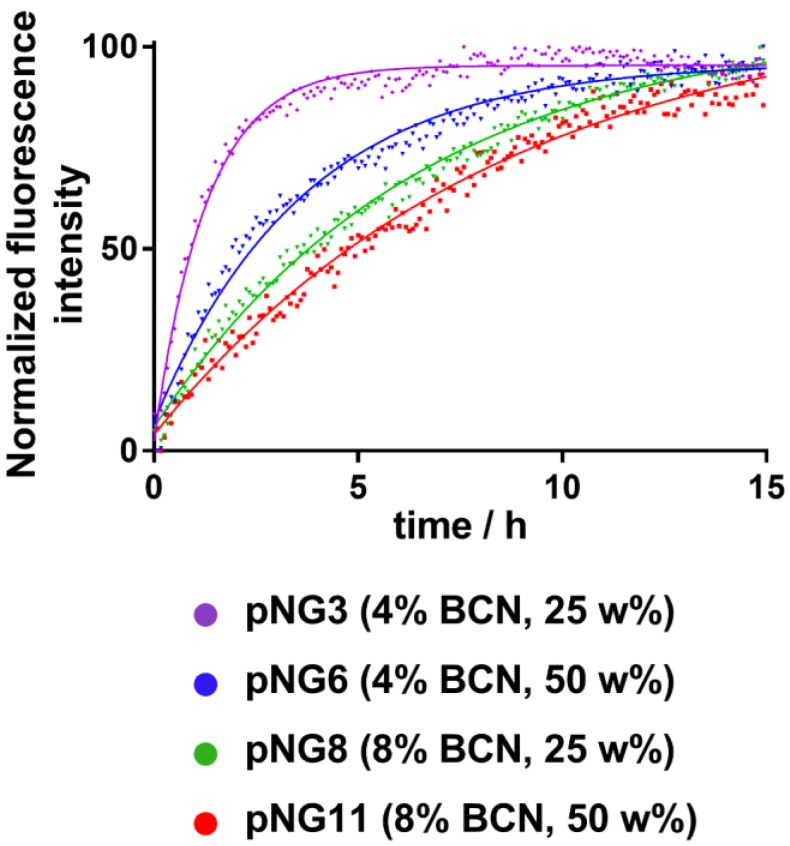
Normalized fluorescence intensity at 405 nm (Ex: 320 nm) over time monitored for different pNGs after addition of MMP‑7 (at t=0) at 37 °C. Lines represent exponential fits used to determine time constants for the degradation process.

The fluorogenic peptide crosslinker was introduced to conveniently follow the degradation of pNGs by fluorescence measurement since upon cleavage of the peptide crosslinks, the fluorescence of the quenched 7-methoxycoumarin (Mca) dye is regained. A library consisting of four pNGs with 4% and 8% BCN functionalization as well as with low and high peptide crosslinker feed were incubated with MMP-7 and the fluorescence intensities of Mca (Em.: 405 nm) were followed over time. The peptide feed and the BCN functionalization in the pNG formation should affect the interior composition. More BCN groups of the nanogels and higher peptide crosslinker feed should result in denser network structure which eventually should be reflected in slower degradation rates. The plot of the fluorescence intensities versus time showed indeed that higher BCN functionalization and constant peptide fraction resulted in slower degradation indicating a denser network structure (Figure 5).

Faster degradation was observed when the fraction of the peptide was reduced during the synthesis suggesting network structures, which are easier accessible for the proteases. Since the number of crosslinking points on the dPG was kept constant, higher peptide fractions can form denser networks which eventually lead to smaller particles as observed in the synthetic screening. The effect of peptide fraction on the degradation rate was more pronounced for pNGs prepared with 4% BCN functionalization. Here, the time constant, which describes how rapidly the degradation process occurs, was nearly increased threefold when the peptide fraction was doubled (1.3 h to 3.7 h), whereas for 8% functionalization, only a minor increase was observed (6.6 Table S3). It can be noted that the non-degradable pNGs did not show an increase of fluorescence signal when incubated with MMP-7 indicating that these pNGs are stable at proteolytic conditions (Figure S4).

Since the pNGs are used as DDS, we were interested to see if cells were able to induce the degradation. Therefore, pNGs were incubated with HeLa cells and the fluorescence intensities of Mca were measured over time. Even though with slower rate, the fluorescence signal was increasing over time suggesting a digestion of the pNGs by cells is feasible (Figure S5).

To exclude media effects and evaluate the stability of pNGs, nanogels were tested in different media including water, PBS, serum-containing cell culture medium, and serum. Degradable and non-degradable nanogels were incubated in the media and the sizes were measured by DLS shortly after mixing and after 24 h. In the DLS measurements, only small changes in sizes were observed indicating that the particles are stable in these media (Figure S6). In cell culture medium, as well as in serum, additional components are present which are detected by DLS and interfere with an accurate measurement. However, since the fluorogenic crosslinker is an indicator of cleavage, it can be used to assess the stability of the pNGs in these media. Therefore, the fluorescence signal of the Mca dye was also monitored over time for different media containing pNGs. Here, no increase in fluorescence intensity was observed either for PBS or for serum-containing medium indicating that the crosslinker is stable under these conditions (Figure S5).

The incorporation of the fluorogenic peptide crosslinker allowed us to study the impact of different feed ratios and the dPG functionalization degree on the degradation rates which translates to different interior compositions of the pNGs. By varying the functionalization of dPG and the fraction of the peptide, the degradation rates showed time constants in the range of about 1 h to 6 h. Hence, we demonstrated that the formation of pNGs with increased feed of peptide crosslinker generated higher crosslinking densities which correspond to the slower cleavage rate. This should allow the tuning of the release kinetics of encapsulated cargo from rather fast to sustained release depending on the desired application.

### Conjugation of Dox to obtain multistage pNGs

Considering the previous data, we chose pNG6 as a suitable candidate for the formation of the multistage DDS because the nanogels presented sizes larger than 200 nm and constant degradation rates with desired MMP-mediated size shrinkage to 40-50 nm fragments. Sizes above 200 nm are suitable for the accumulation process in tumor tissue following the EPR effect and additionally, should avoid internalization of the nanogels into normal and malignant cells 59. Only after size shrinkage in the tumor microenvironment, the pNGs fragments can be readily taken up by the malignant cells where the chemotherapeutic drug can be released. To realize the covalent conjugation of therapeutic agent through a pH-sensitive linkage, we chose a known prodrug of the anticancer drug DOX, namely aldoxorubicin. This prodrug comprises a maleimide group readily reacting with free thiols. This thiol-binding DOX derivative was designed to hitchhike albumin as carrier in the blood stream by probing a specific cysteine of the serum protein 60-62.

After the crosslinking process, the pNGs were easily modified by quenching the excess of BCN groups with an azide-functionalized cyanine dye (ICC-azide) to label the pNGs or with 1-Azido-4,7,10-trioxa-13-tridecanamine (N_3_-TOTA) to introduce amine groups. The ICC label is used to follow the pNGs and the degradation fragments in the following diffusion and penetration experiments. After purification, the free amine groups were thiolated *in situ* using 2-iminothiolane. The formed thiols readily reacted in a Michael addition reaction with the maleimide groups of aldoxorubicin yielding multistage pNGs (Figure 2). The approach of *in situ* thiolation was chosen to avoid crosslinking of the reactive precursors. As before, non-degradable control pNGs were prepared using a peptide crosslinker synthesized with d-amino acids. The hydrodynamic diameters were barely affected by the modification of the pNGs with the drug, but the slightly positive surface charge was marginally increased by the attachment of aldoxorubicin HCl salt. The DOX contents of degradable and non-degradable pNGs were determined by UV/Vis spectroscopy with 1.8 w% and 2.0 w%, respectively (Figure S7a+b). All synthesized pNGs had similar sizes and dye/drug loadings allowing to compare their potential to increase the penetration efficiency and their therapeutic activity (Table 1).

The acid-mediated release of DOX from the pNGs was confirmed by incubation of the multistage pNGs at pH 7.4 and pH 5 as well as in the presence of MMP-7. Here, we found that the release of DOX is accelerated in acidic pH. In the first 24 h, ~50% of DOX was released at pH 5. In earlier studies, we reported a release of 60% at pH 5 for aldoxorubicin-derived polymer-drug conjugates 63, 64. The slightly lower release of the pNG-Dox system is probably caused by drug-carrier interaction within the nanogel network. Therefore, an increase in release is expected when the pNGs are first digested with MMP and subsequently, exposed to acidic pH values. Indeed, under these conditions the release was increased to ~60% (Figure S8). In the mentioned polymer-drug conjugates, release at pH 7.4 was marginal with less than 10% over several days. For the nanogels however a release of ~25% was observed at pH 7.4 indicating that DOX is not only covalently linked but also partially encapsulated, and therefore can leak out of the nanogel network by diffusion. Even intensive purification by dialysis and SEC column after the conjugation step did not change the release profile suggesting that a small fraction of encapsulated DOX always remained in the network.

### Diffusive transport in agarose matrix

**Figure 6:**
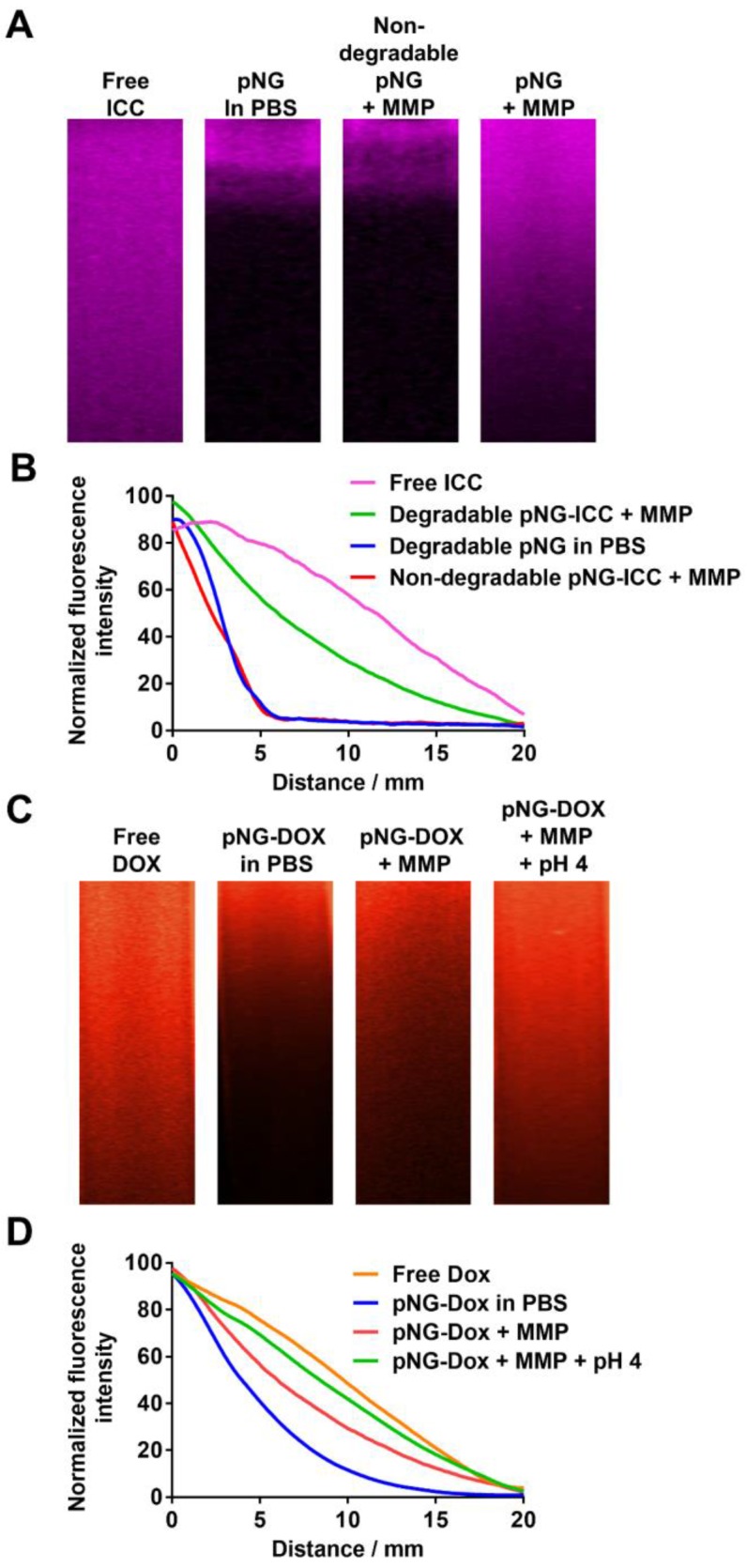
a) Diffusion of free ICC and ICC-labeled pNGs in agarose gel before and after incubation with MMP-7. b) Normalized intensity profiles of free ICC and ICC-labeled pNG in agarose gel. c) Diffusion of free DOX and pNG-DOX in agarose gel before and after incubation with MMP-7 and at acidic pH. d) Normalized intensity profiles of free DOX and pNG-DOX in agarose gel.

The potential of pNGs fragmentation by MMPs to enhance diffusive transport was studied by the penetration of digested and undigested pNGs in dense agarose matrix mimicking the dense ECM in tissue. For this, we first brought solutions of ICC-labeled pNGs and the non-degradable control pNGs, before and after incubation with MMP-7, in contact with the agarose gel and incubated for 16 h at 37 °C. Before digestion, degradable and non-degradable pNGs presented negligible penetration into agarose gel. However, after incubation with MMP-7, the smaller fragments of the degradable nanogels were able to penetrate deep into the agarose matrix (Figure 6a) whereas the non-degradable control did not show any penetration. The intensity profiles of the fluorescence signal in the gel were plotted in Figure 6b. The signals for non-digested particles and for non-degradable control pNGs incubated with MMP-7 descends to 50% of the initial value after ~2.5 mm, whereas the intensity of digested pNGs was reduced to 50% of the initial value only after 8.9 mm indicating that the penetration is enhanced.

Having confirmed an increased diffusion after pNG degradation using dye-labeled nanogels, we investigated the performance of the multistage DDS, pNG-DOX, in agarose gel diffusion. In this case, the intrinsic fluorescence of DOX allowed us to follow the infiltration. Before the incubation with MMP only marginal penetration of DOX was observed (50% after 4.0 mm), whereas after incubation with MMP, the particles diffused into the gel matrix (50% after 6.8 mm; Figure 6c+d).

To emulate the second step in the multistage delivery—the pH-dependent release of DOX from dPG by cleavage of the linking hydrazone bond—we further incubated the digested pNGs at acidic pH. Penetration of DOX into the agarose matrix was notably increased to 50% after 8.3 mm. This experiment confirmed an improved diffusion efficiency for degradable pNGs indicating that the fragmentation of the pNGs could improve the penetration into the interstitial matrix of tumor tissue.

### Penetration into multicellular tumor spheroids

To confirm that the MMP-induced size reduction of pNGs can enable penetration into tumor tissue, we employed multicellular tumor spheroids to test the hypothesis in a tumor resembling 3D model. MCTS capture complex 3D tissue physiology such as presence of ECM as well as pH, oxygen, metabolic, and proliferative gradients and provide a useful technique to study anticancer strategies *in vitro* 65-69. In particular; diffusion-based transport of nanoparticles in an environment that resembles the structural and microenvironmental conditions associated with solid tumors is of great interest 68, 70. For modeling the cellular diversity in tumor tissues, a variety of spheroid-based co-culture systems have been developed, e.g., with fibroblasts and endothelial cells 71. Among the major cell types that are pivotal for conditioning the microenvironment are fibroblasts 72. This infiltrating cell type is known to be coopted by tumor cells to promote invasion to other tissue, and thereby contributes to the formation of metastasis 73, 74. In particular, fibroblasts contribute to tumor invasiveness by producing MMPs which regulate the remodeling of the surrounding ECM 73, 75. Therefore, co-cultures of cancer cell lines and fibroblasts enable the formation of 3D spheroids models that resemble the tumor physiology or microenvironment and provide the proteases necessary to degrade the pNGs.

To grow MCTS, HeLa cells and primary fibroblasts at different ratios and cell numbers were screened to reproducibly obtain spheroids with sizes of ~500 µm. For a mixture of HeLa and fibroblasts in ratio 2:1, spheroids were obtained after 4 days and further grew to dense circular structures with sizes of approximately 500 µm after 7-9 days (Figure S9).

**Figure 7:**
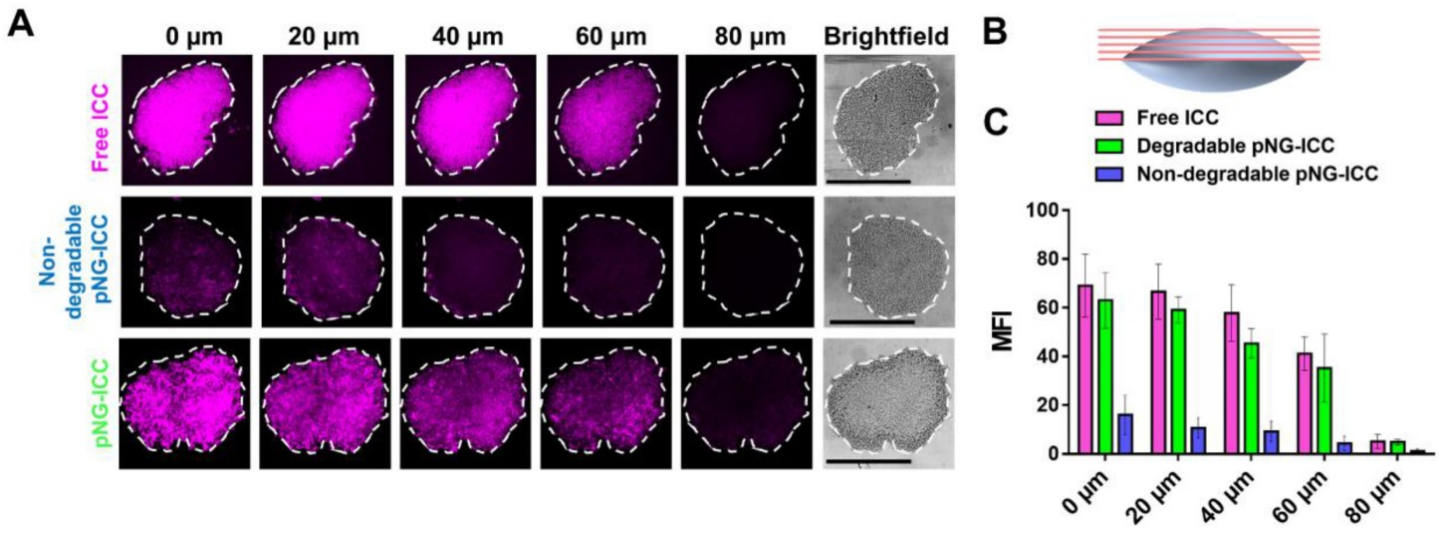
a) Penetration of free ICC, degradable, or non-degradable pNGs labeled with ICC into MCTS. The black bars in the brightfield images represent 500 µm; CLSM images with 20-fold magnification. b) Schematic representation of the flattened spheroids and the optical sectioning. c) Mean fluorescence intensities of ICC in the area of the MCTS for increasing depth.

To investigate the diffusion properties of the DDS in MCTS, we first used the dye labeled pNGs. MCTS with sizes of around 500 µm were treated with ICC-labeled degradable (pNG-ICC) and non-degradable pNGs (non-degradable pNG-ICC). In the first set of experiments, spheroids were washed after incubation with the compounds for 16 h, transferred to a µ-slide, and the living MCTS were directly imaged using a CLSM. The z-stack function was used to optically section the spheroids in 40 µm steps. Here, we could indeed observe that the free dye and degradable pNGs yielded higher fluorescence intensity in deeper layers compared to the non-degradable control. However, when the images were compared with the brightfield image, it appeared that only the surface of the spheroids was imaged because the laser light is absorbed by the tumoroid tissue resulting in a limited penetration depth (Figure S10). To compensate the absorption of the laser light in large objects, the spheroids were mounted between microscope slide and cover glass and were thereby slightly squeezed (Figure 7b). These mounted MCTS allowed to image the central region of the spheroids by optical sectioning in the confocal microscope since the laser light does not need to penetrate into deeper layers. We found that the free dye used to label the pNGs had penetrated to the interior of the MCTS to a point where the signal abruptly decreased (Figure 7a), because the light absorption of the tissue becomes too large to obtain proper signals.

Nevertheless, optical sections of spheroids treated with the degradable control displayed an efficient penetration into the spheroid. Compared to the free dye, the distribution of fluorescence signal is not as homogeneous for the degradable pNGs indicating that larger fragments may accumulate in more spacious regions of the spheroid. A distinct difference is apparent in comparison to the non-degradable control. Here, all sections displayed a low intensity and a marginal signal was observed in deeper layers confirming that the non-degradable pNGs are not able to diffuse into the MCTS. The graph in Figure 7c shows the mean fluorescence intensity (MFI) over the area of the spheroid section illustrating that free dye and the degradable nanogel display similar intensities over the total area of the section with slow decrease for deeper sections.

**Figure 8:**
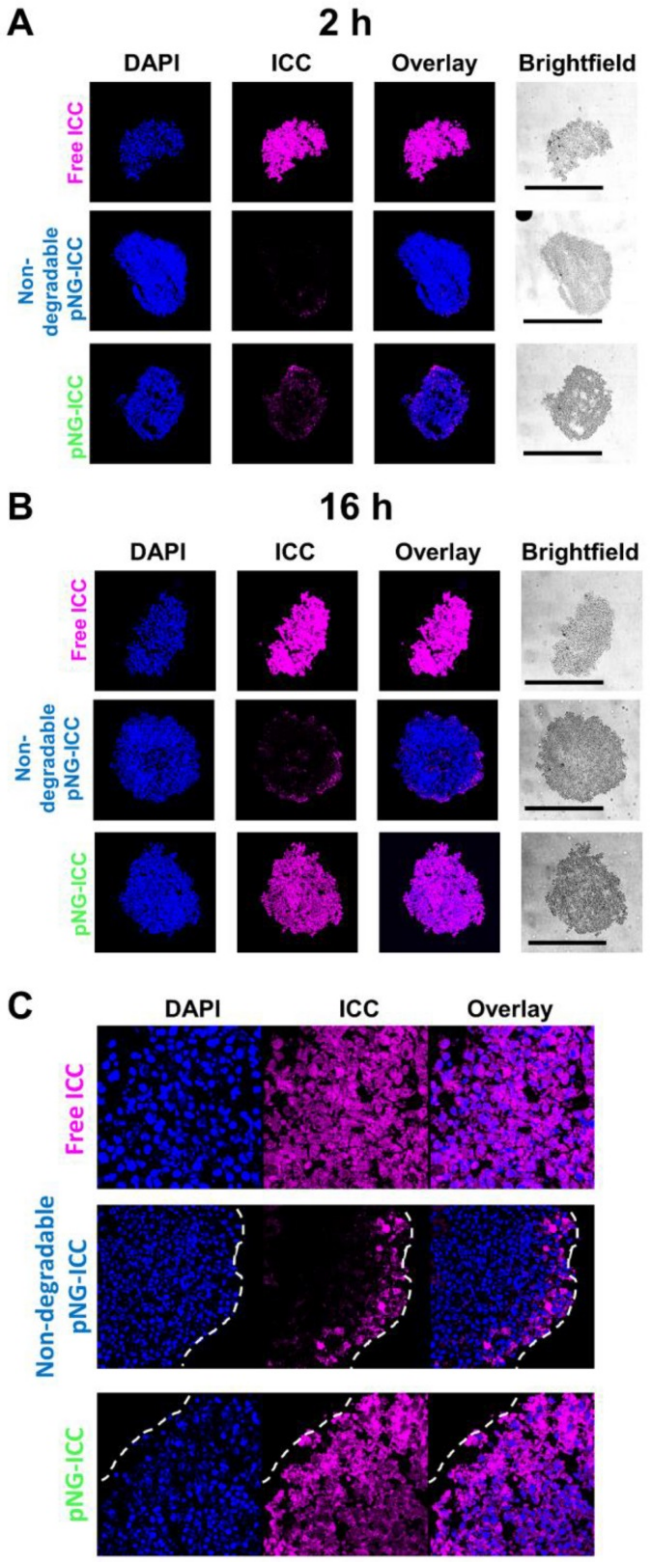
CLSM images of MCTS cryosections with 20-fold magnification. MCTS were incubated with free ICC, degradable, or non-degradable pNG-ICC for a) 2 h and b) 16 h, respectively. The black bars in the brightfield images represent 500 µm. Mean fluorescence intensities over the area of spheroid sections are shown in the SI (Figure S11). c) CLSM images of cryosections with 64-fold magnification.

To confirm the results obtained from optical sections, cryosections of the spheroids incubated with labeled pNG-ICC for 2 h and 16 h were prepared. Representative cryosections from the mid-region of spheroids were imaged by fluorescence microscopy. Free ICC and DAPI were found to be homogeneously distributed throughout the sections at both time points indicating an unhindered diffusion of the free dyes through the spheroid (Figure 8a). For the degradable pNGs and the non-degradable control, only minor intensities were observed at the margins of the spheroids after 2 h. For longer incubation time, we found minimally increased fluorescence intensity when treated with the non-degradable pNGs. At the periphery, intense signals were observed, however, the intensity was rapidly declining over the first 20 to 30 µm towards the center of the section. When the degradable pNGs were incubated for 16 h with the MCTS, intense fluorescence was observed throughout the section area (Figure 8b + S11). The sections showed a gradient with higher fluorescence intensity at the periphery that decreased towards the core which is apparent from the images with higher magnification (Figure 8c). It is notable that the core still possessed distinct fluorescence intensity suggesting that the degradation products penetrated to the core of the spheroids. These observations support the results of the optical sections and confirm that the degradability of the pNGs are a favorable property for an efficient penetration of the tumor-resembling 3D model.

Since we have shown before that the fragmentation of the pNGs is caused by proteases (Figure 4), we can assume that the increased distribution of the fluorescence signal associated with the nanogels and their fragments resulted from the degradation of the pNGs by expressed proteases. It has been reported that MMPs are indeed expressed in *in vitro* scenarios and in particular in co-cultures using primary cells 76, 77.

**Figure 9:**
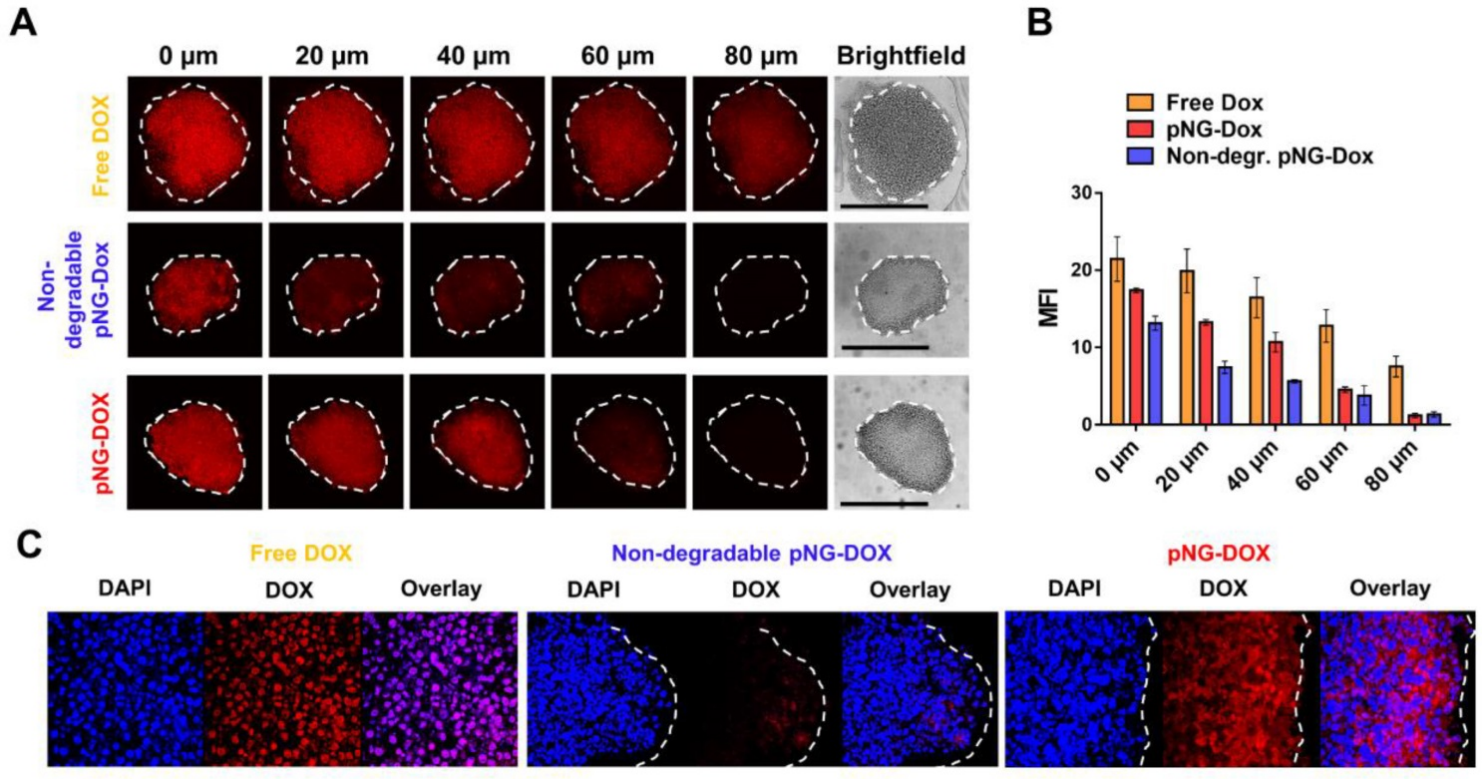
a) Penetration of pNG-DOX into MCTS: Comparison of free DOX, degradable multistage pNG-Dox, and the non-degradable control. The black bars in the brightfield images represent 500 µm; CLSM images with 20-fold magnification. b) Mean fluorescence intensity of DOX over the area of the MCTS for different penetration depth. c) CLSM images of cryosections with 64-fold magnification.

To demonstrate that the results obtained from the dye-labeled pNG can be transferred to the performance of the multistage pNG-Dox, spheroids were incubated with the multistage pNG-Dox, free DOX and the non-degradable control. After incubation, we could see that the free drug was distributed homogeneously throughout the spheroids with slightly decreasing fluorescence intensity for deeper regions. In comparison, the penetration for the degradable pNG-DOX was considerably higher than for the non-degradable control especially for deep sections of the tumor spheroids (Figure 9a). This indicates that pNGs are degraded and that the small fragments possessed an advantage in penetrating into deep regions of the 3D tumor model. Comparing to the penetration study using dPG-ICC, it can be noted that the DOX penetration for the non-degradable system was higher than for the non-degradable dPG-ICC (Figure 9b). This can be explained by either premature DOX release or diffusion of small fractions of encapsulated DOX.

To complement this data, cryosections of MCTS incubated with pNG-DOX and controls were prepared for 2 h and 16 h (Figure S12). For the degradable dPG-Dox a time dependent increase in penetration depth of DOX fluorescence was observed, whereas for the non-degradable control no change was visible over time. The confocal images at higher magnification illustrate the enhanced penetration of DOX for the degradable pNGs (Figure 9c). These observations support the results of the optical sections and confirm that the size reduction property of the pNGs enhances the transport of the therapeutic agent into the tumor-resembling 3D model.

Furthermore, the cryosections of the spheroids give information about the fate of the nanogels (Figure 8c and 9c). Here, it can be recognized that non-degradable nanogels are not localized in the cytosol but rather in the extracellular space. On the other side, the signal of samples with degradable nanogels can be found intracellularly. The observation supports the assumption that the intact nanogels with sizes larger than 200 nm are internalized less by cells 59. Only after degradation, smaller fragments are readily internalized and able to release their therapeutic cargo in acidic compartments. This should result in a different therapeutic activity of DOX when transported by degradable or non-degradable nanogels which is discussed in the next part.

### Therapeutic activity of multistage pNG

**Figure 10:**
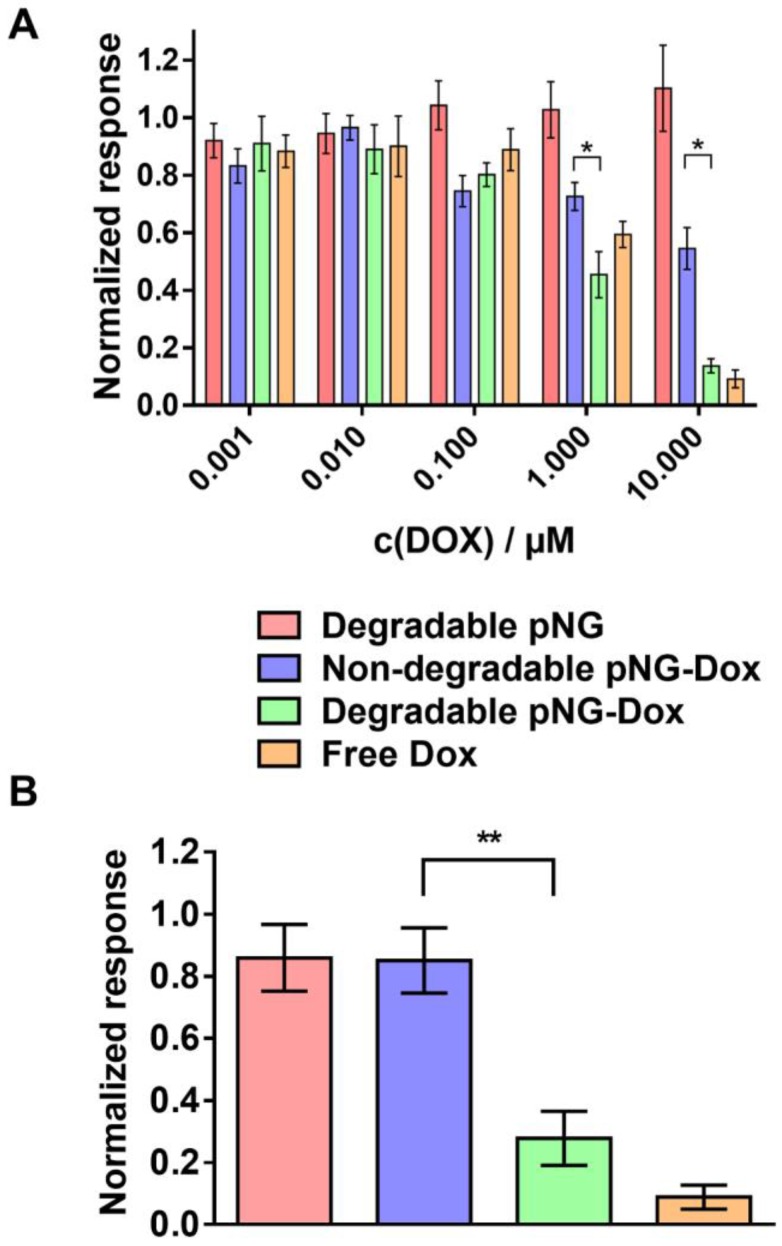
Therapeutic activity of DOX in the multistage DDS. a) Cell viability after treatment with different concentration of pNG-DOX and controls in monolayer culture of HeLa cells. b) Cell viability after treatment with pNG-DOX and controls at 10 µM DOX concentration in MCTS model after 48 h of treatment, respectively. Error bars indicate SEM from three independent measurements; untreated cells = 1.0 response; stars indicate statistical significance between degradable and non-degradable pNGs determined by non-parametric t-test (* p < 0.1;** p < 0.05).

To confirm that DOX is still therapeutically active after degradation of the pNG and tissue penetration, we analyzed the activity of DOX on cell viability after incubation with pNGs in monolayer culture as well as in MCTS. For the monolayered culture, the viability was determined by the ability of the cells to metabolize MTT. The bare pNGs did not display any toxicity towards HeLa cells up to the highest concentration tested (0.25 mg/mL), whereas free DOX and the multistage pNG-DOX reduced the viability of HeLa cells significantly starting at 1 µM DOX concentrations (Figure 10a).

Interestingly, minor toxicity towards HeLa cells was observed for the non-degradable control indicating that the degradability of the pNGs plays a crucial role for the therapeutic activity. Since the release of DOX for the pNGS was marginal at pH 7.4 but increased under acidic conditions as found in intracellular compartments this result suggest that the internalization of non-degradable pNG is suppressed and that the small fragments of the degradable pNGs can be taken up more readily. This observation is supported by the magnified images of the spheroid cryosections. Here, the fluorescence signals associated with the digested pNGs (ICC, Figure 8c) and the drug (Figure 9c) are localized in intracellular compartments for the degradable pNGs whereas the signals for the non-degradable sample is mainly found in the extracellular space. This supports our assumptions that the intact nanogels are internalized less by the cells adding a targeting component to the multistage pNGs.

We were interested to see if the difference between degradable and non-degradable pNGs in 2D cell culture can also be observed in the treatment of the 3D model with pNGs. After incubation of the spheroids with the pNGs, the viability of cells in spheroids was assessed by measuring intracellular adenosine triphosphate (ATP) content using the CellTiter-Glo^®^ assay because only low signals were obtained with MTT. Here, the non-degradable control marginally inhibited the proliferation of the spheroids when treated with a DOX concentrations of 10 µM. In contrast, when treated with the degradable pNG-DOX, ATP concentrations were reduced to 22% relative to the untreated control (Figure 10b). The difference emphasizes that the degradation-induced penetration is crucial for the treatment of the tumor model to deliver the drug to the inner regions. Therefore, we suggest the two-stage strategy of the presented multistage pNGs as potential nanocarrier-based DDS for systemically applied drug delivery. After extravasation to the tumor tissue, the protease-mediated fragmentation could enhance the tumor penetration to promote uniform drug distribution.

## Conclusion

In summary, we prepared a novel MMP- and pH-sensitive multistage delivery system in the form of pNGs. The incorporation of a smart fluorogenic peptide crosslinkers into a hydrophilic, dPG-based scaffold provided degradability into synthetic NGs comprised of otherwise non-degradable polymers. The size reduction of these particles can be triggered by an endogenous stimulus in the tumor microenvironment, and thereby promotes the penetration of polymer-drug conjugates into dense tissue. The pNGs were prepared by surfactant free inverse nanoprecipitation using strain-promoted click chemistry. The procedure yielded nanogels with spherical morphology and good to excellent size distribution. The variation of BCN functionalization on dPG and the fraction of the peptide crosslinker allowed control over the size and the degree of crosslinking. The size reduction property of the pNGs in the presence of MMP was demonstrated by time dependent size measurements showing the desired reduction from several 100 nm to sub-50 nm fragments. The MMP-mediated degradation was studied in detail by fluorescence measurements following the fluorescence recovery of the intrinsic reporter moiety of the crosslinker. Here, we found that the degradation rate depends on the feed ratio and BCN functionalization with slower rates for higher crosslinker feed. This feature could be used to tune the release rate of an encapsulated cargo. The pNGs were post-synthetically modified to covalently attach the chemotherapeutic drug DOX through an acid-sensitive hydrazone linkage. The size and surface charge were barely affected by the modification, and the release of DOX from the pNGs was enhanced at acidic conditions. The digested multistage pNGs showed enhanced diffusive transport through a dense gel matrix and we successfully demonstrated in tumor resembling MCTS models that the size-changing property of the pNGs promotes the infiltration of the functional chemotherapeutic drug into deeper tissue regions. Therefore, the multistage dPG constitutes a potential nanocarrier for systemic application to promote drug delivery in solid tumors. Given the difficulty to deliver chemotherapeutics homogeneously to the dense center of solid tumors, the developed multistage peptide-crosslinked nanogels present a promising design for efficient drug delivery to malignant tissue. Further, the facile and smart design provides unprecedented insights into the composition and degradation kinetics of the nanogels *in situ* due to the intrinsic reporter unit. The intrinsic reporter and the controlled release of a chemotherapeutic drug emphasize the theranostic character of the nanogel design.

## Supplementary Material

Supplementary information, experimental data, figures, and tables.

## Figures and Tables

**Table 1:** Physicochemical characterization of multistage pNGs.

Sample	Peptide linker	Size*^a)^* (DLS) [nm]	PDI (DLS)	ζ-potential*^b)^* [mV]	Functionalization*^c)^*[w%]
pNG	cleavable	214 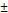 4.9	0.117	+4	-
pNG-DOX	cleavable	218 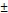 3.9	0.220	+9	1.8
pNG-ICC	cleavable	244 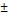 4.8	0.105	+2	0.8
pNG	non-cleavable	193 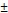 1.7	0.043	+5	-
pNG-DOX	non-cleavable	220 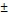 3.4	0.100	+9	2.0
pNG-ICC	non-cleavable	212 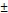 1.5	0.026	+3	0.8

^a)^ Mean hydrodynamic diameter and standard deviation from three measurements obtained by DLS in H_2_O at 25 °C. Intensity distribution is given; ^b)^ For ζ-potential measurements, electrophoretic mobility of the pNGs was analyzed following application of a 20 Vcm^-1^ electric field; ^c)^ Determined by UV/Vis spectroscopy using the extinction coefficient of the ICC dye and DOX, respectively.

## Abbreviations

ATP: adenosine triphosphate
BCN: bicyclononyne
CLSM: confocal laser scanning microscope
dPG: dendritic polyglycerol
DMF: *N,N*-dimethylformamide
Dnp: (2,4-dinitrophenyl)
DOX: doxorubicin
DDS: drug delivery system
DMEM: Dulbecco's Modified Eagle Medium
DLS: dynamic light scattering
EDTA: ethylenediaminetetraacetic acid
EPR: enhanced penetration and retention
EG: ethylene glycol
ECM: extracellular matrix
FBS: fetal bovine serum
FRET: fluorescence resonance energy transfer
ICC: indocarbocyanine
MMP: matrix metalloproteinase
Mca: 7-methoxycoumarinyl-4-acetic acid
MWCO: molecular weight cut-off
MCTS: multicellular tumor spheroids
NTA: nanoparticle tracking analysis
pNGs: peptide-crosslinked nanogels
PBS: phosphate-buffered saline
PDI: polydispersity index
SEM: standard error of the mean
TEM: transmission electron microscopy
