# Supplementary material for: Matrix Metalloproteinase-sensitive Multistage Nanogels Promote Drug Transport in 3D Tumor Model
Source: Theranostics. 2020 Jan 1;10(1):91–108. doi: 10.7150/thno.34851 (PMC6929628; doi:10.7150/thno.34851)

Supporting information

Matrix metalloproteinase-sensitive multistage nanogels enhance drug transport in 3D tumor model

*Gregor Nagel[1], Ana Sousa-Herves[1], Stefanie Wedepohl[1], Marcelo Calderón[1,2,3]**

[1]*Freie Universität Berlin, Institute of Chemistry and Biochemistry, Takustr. 3, 14195 Berlin, Germany.*

[2]*POLYMAT and Applied Chemistry Department, Faculty of Chemistry, University of the Basque Country UPV/EHU, Paseo Manuel de Lardizabal 3, 20018 Donostia-San Sebastián, Spain*

[3]*IKERBASQUE, Basque Foundation for Science, 48013 Bilbao, Spain.*

*Corresponding author:

E-mail: marcelo.calderon@fu-berlin.de; marcelo.calderon@polymat.eu

**Table of content:**	**Page**
**I. General information**	**S3**
**II. Figures and Schemes**	**S6**
**III. Experimental data**	**S14**

I. General information

Plate reader

After samples and protease were added, the microplates (Sarstedt or Brand) were covered with optically clear adhesive seal sheets (Absolute QPCR Seal, Thermo Scientific) and placed into a Tecan Infinite M200 Pro microplate reader heated to 37 °C. Fluorescence of methoxy coumarine was monitored every 5 min over 16 h using an excitation wavelength of 320 nm (9 nm bandwidth) and an emission wavelength of 405 nm (20 nm bandwidth).

GPC

Gel permeation chromatography (GPC) analysis were performed on a Shimadzu Prominenze-i LC-2030 liquid chromatography system equipped with a Shimadzu RID-20A refractive index detector. The GPC column used was a Shodex OHpak SB-806M HQ with OHpak SB-G 6B as guard column. Solvents with HPLC grade by Fisher Chemical were employed. The oven temperature was set to 30 °C. The method included a flow 0.5 mL/min with an isocratic mobile phase (PBS 10 mM phosphate, 50 mM NaCl, pH 7.4). The injection volume was 50 µL and the UV-detectors were set to 490 and 650 nm. GPC data was analyzed by Shimadzu LabSolution Version 5.85 software.

Dynamic light scattering (DLS) and zeta potential

Size distribution and zeta potential of pNGs were measured at 25 °C by dynamic light scattering (DLS) using a Zetasizer Nano-ZS 90 (Malvern) equipped with a He-Ne laser (λ = 633 nm) at a scattering angle of 173°. Samples with the concentration of 1 mg/mL in water equilibrated for 5 min at the respective temperature prior to the measurement. Particle size distributions are given as the average of three measurements from intensity distribution curves. As DLS measurements of

the NGs were monomodal in distribution, with autocorrelation functions showing a single exponential decay. The autocorrelation functions of backscattered light were analyzed using the Zeta-sizer DTS software from Malvern to determine the size distribution by intensity and the polydispersity index The hydrodynamic diameters are reported from the intensity distribution curves.

Transmission electron microscopy (TEM)

Transmission electron microscopy samples were prepared by blotting samples (1 mg/mL) onto carbon-coated copper grids (400 meshes, Quantifoil Micro Tools GmbH). Then a droplet (5 µL) of 1% (w/v) uranyl acetate solution was applied and kept for 60 s before the excess of contrasting material was removed by means of filter paper and the sample could dry in air. Samples were visualized by using the TEM detector on a Hitachi scanning electron microscope (SU8030, Hitachi, Tokyo, Japan) at 20–30 kV and 10 µA at different magnifications.

MCTS culture

HeLa cells or HeLa/fibroblast cell mixtures were cultured in hanging drops using the GravityPLUS™ kit (InSphero AG). According to the manufacturer's instructions, drops of 40 µL at various cell densities ranging from 500 to 10000 cells per drop were seeded to observe the formation of spheroids of at least 500 µm in diameter. After 3 d at 37 °C and 5% CO_2, spheroids grew on the bottom of most of the drops. The spheroids were transferred to GravityTRAP™ plates, a non-adhesive coated 96-well microplate with conic wells for longtime cultivation, by adding 70 µL media to the drops and subsequent centrifugation of the plate at 300 rpm for 2 min to force the spheroids into the wells. The spheroids were stored at 37 °C and 5% CO_2. Medium was exchanged once per week. Between day 7 and 9 after seeding, spheroids reached a size of approximately 500–600 µm and showed dense circular structures and were ready to use for the penetration assay (Figure S8).

Cell viability assay

To assess cell viability and proliferation inhibition, 10000 cells per well were seeded into 96-well-plates (Sarstedt) with 100 µL of culture medium (RPMI for HeLa, DMEM for all other cell lines) with 10% FBS (FBS Superior, Merck), 1% Penicillin/Streptomycin (Thermo Fisher Scientific). The cells were incubated at 37 °C at 5% CO_2 overnight. Then, the media was replaced with fresh media containing various dilutions of the corresponding NG or free drug in duplicates and cells were incubated for 48 h at 37 °C and 5% CO_2. The cell culture supernatant was removed, and cells were washed twice with PBS (200 µL/well). Then, 100 µL/well fresh full medium including 10 µL/well MTT (Sigma-Aldrich, 5 mg/mL in PBS) were added and incubated for another 4 h at 37 °C. After development of formazan crystals, the cell culture supernatant was removed, and crystals were dissolved by addition of 100 µL/well of isopropanol containing 0.04 M HCl. Absorbance was read at 590 nm in a Tecan Infinite M200 Pro microplate reader. In case of the 3D-model, spheroids were incubated with the corresponding pNGs or the free drug with DOX concentrations of 10 µM in triplicates for 48 h at 37 °C and 5% CO_2. Afterwards, CellTiter-Glo® (Promega corporation) viability assay solution was added and the contents were mixed for 5 min to induce lysis of the cells. The plate was incubated for 10 min at rt to stabilize the luminescence signal and luminescence was recorded in the plate reader. Relative viabilities were calculated by dividing average absorbance or luminescence values of wells with treated cells by values of untreated cells (=100% viability). All tests were repeated 3 times independently and errors were expressed as standard error of the mean (SEM).

II. Figures and Schemes

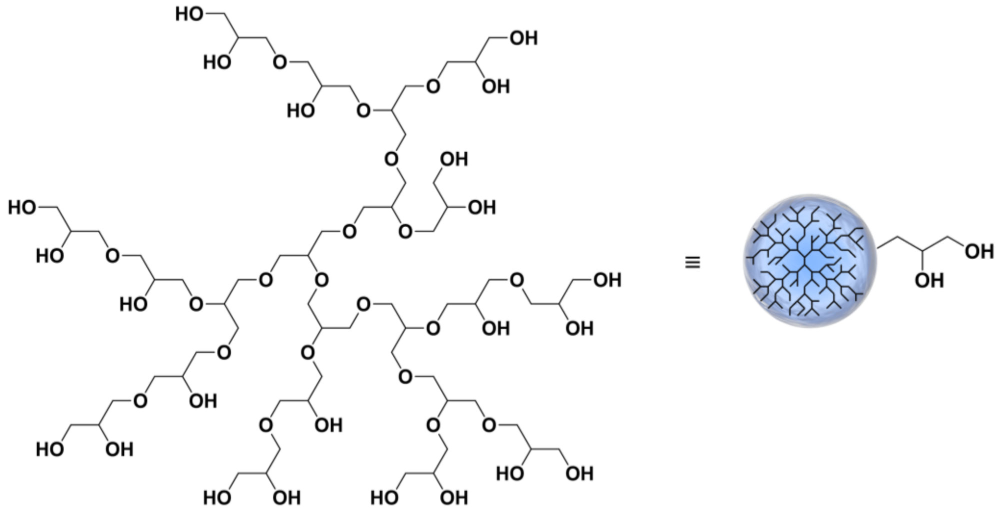

Figure S1. Representative structure of dendritic polyglycerol (dPG).

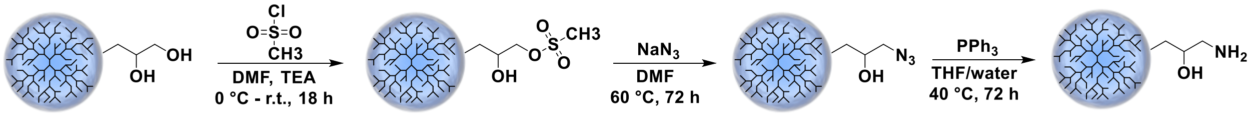

Scheme S1. Functionalization of dPG with amine groups in three steps.

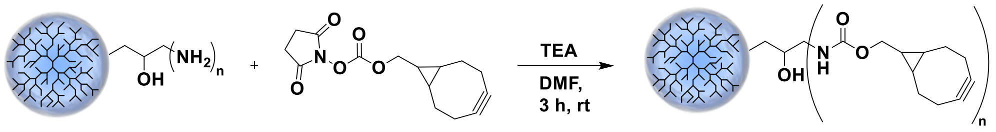

Scheme S2. Functionalization of dPG with bicyclo[6.1.0]non-4-yn (dPG-BCN).

Table S1: Composition of feed and resulting hydrodynamic diameters of prepared pNGs.

#	dPG-BCN (%OH groups converted)	Peptide crosslinker [w%]	Peptide crosslinker [mol%]	Reactant concentration [mg mL^{-1}]	size[a] in H_2O [nm]	Poly dispersity index
**pNG 1**	4	10	20	3.0	676	0.239
**pNG 2**	4	20	25	3.0	414	0.345
**pNG 3**	4	25	35	3.0	308	0.128
**pNG 4**	4	40	50	3.0	270	0.205
**pNG 5**	4	45	70	3.0	254	0.150
**pNG 6**	4	50	80	3.0	214	0.117
**pNG 7**	4	70	105	3.0	178	0.175
**pNG 8**	8	25	17.5	3.0	635	0.077
**pNG 9**	8	40	35	3.0	543	0.030
**pNG 10**	8	45	40	3.0	502	0.108
**pNG 11**	8	50	50	3.0	379	0.032
**pNG 12**	8	70	60	3.0	367	0.314
**pNG 13**	4	45	70	2.0	121	0.324
**pNG 14**	4	45	70	4.0	270	0.023
**pNG 15**	4	45	70	8.0	426	0.349

[a] Mean hydrodynamic diameter obtained by dynamic light scattering (DLS) measurements in H_2O at 25 °C. Intensity distribution is given

Table S2. Hydrodynamic diameter determined by DLS of degradable and non-degradable pNGs in different media. The mean of three measurements and the respective standard deviations are depicted.

Media	Degradable pNGs [nm]	Non-degradable pNGs [nm]
Water	386 ± 9.6	310 ± 10.2
PBS (TCNB[a)])	289 ± 2.1 (286 ± 11.5)	247 ± 2.4 (248 ± 5.0)
Cell culture media (RPMI)	295 ± 7.3	242 ± 5.1
Serum[b)]	289 ± 8.2	228 ± 3.5

a) TCNB – 50 mM Tris base, 10 mM CaCl2, 150 mM NaCl, 0.05% (w/v) Brij-35, pH 7.5; b) 10% in PBS

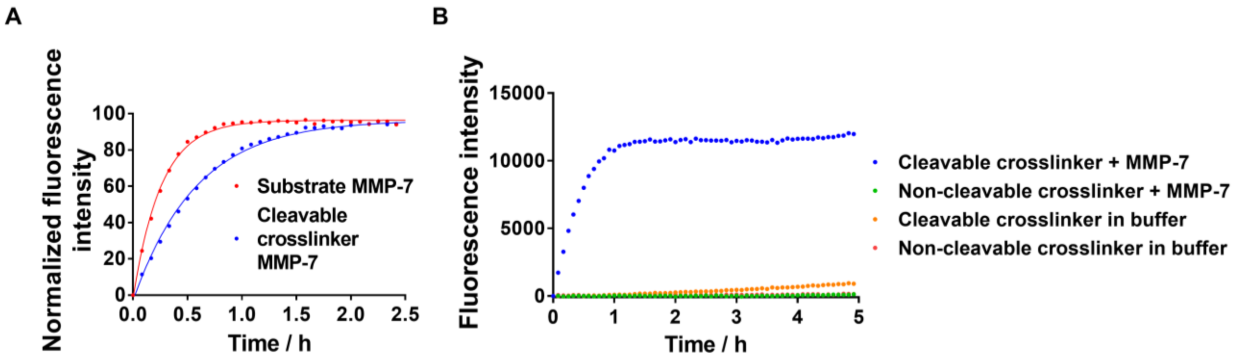

Figure S2. (A) Change of fluorescence intensity (Excitation (Ex): 320 nm; Emission (Em): 405 nm) over time for a commercial substrate and cleavable fluorogenic peptide crosslinker incubated with matrix metalloprotease 7 (MMP-7) at 37 °C. Time constants: Substrate 0.26 h; crosslinker: 0.58 h with same concentration. (B) Change of fluorescence intensity (Ex: 320 nm; Em: 405 nm) over time for cleavable and non-cleavable fluorogenic peptide crosslinker incubated in buffer or in the presence of MMP-7 at 37 °C.

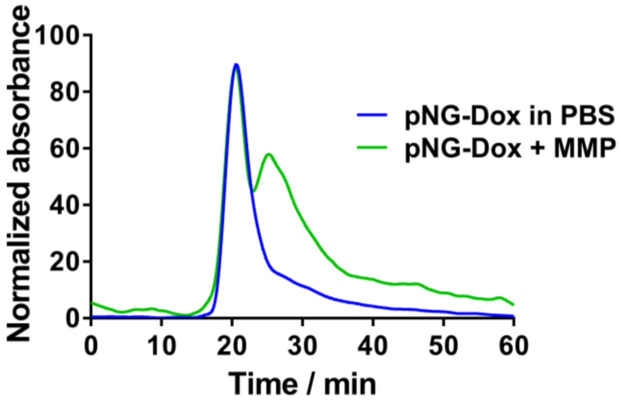

Figure S3. Normalized gel permeation chromatography traces of multistage pNG-Dox before and after incubation with MMP-7 for 16 h.

Table S3: Time constants of degradation rate determined from the exponential fit of the fluorescence intensities over time.

pNG	Time constant (1/K; h)
pNG3	1.3
pNG6	3.7
pNG8	6.6
pNG11	8.5

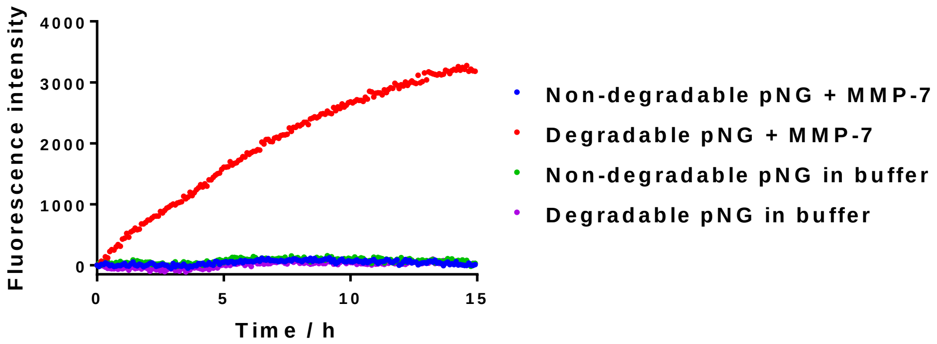

Figure S4. Change of fluorescence intensity (Ex: 320 nm; Em: 405 nm) over time for peptide-crosslinked nanogels (pNGs) incubated in buffer or in the presence of MMP-7 at 37 °C.

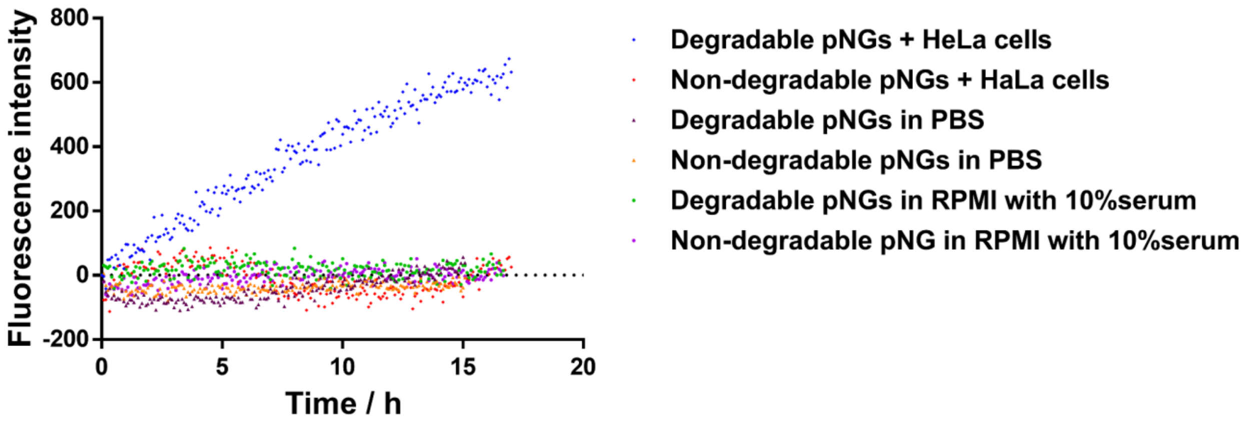

Figure S5. Change of fluorescence intensity (Ex: 320 nm; Em: 405 nm) over time for degradable and non-degradable pNGs incubated with Hela cells (10^5 cells/mL), Tris-buffered saline or serum-containing cell culture medium (10% FBS in RPMI) at 37 °C.

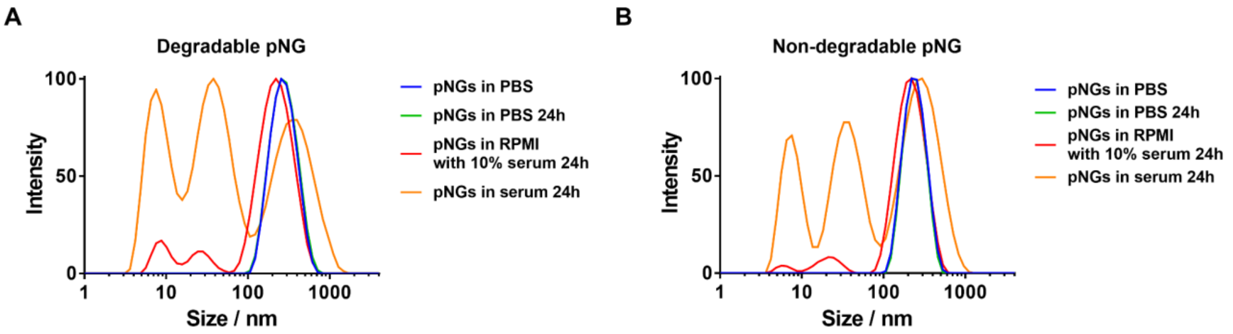

Figure S6. Size measurements by DLS of (A) degradable and (B) non-degradable pNGs after incubation in different media.

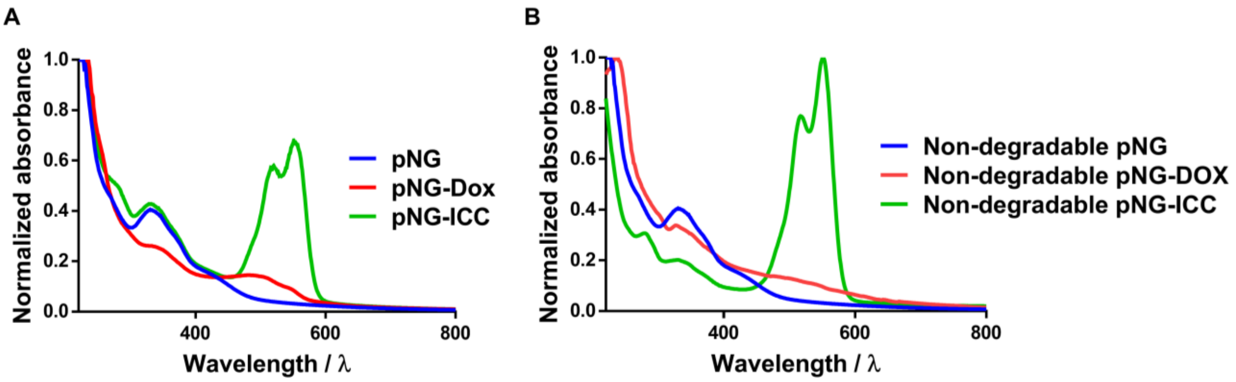

Figure S7. UV/Vis-spectra of (A) degradable and (B) non-degradable pNGs including unfunctionalized pNGs, multistage pNG-Dox, and labeled pNG-ICC.

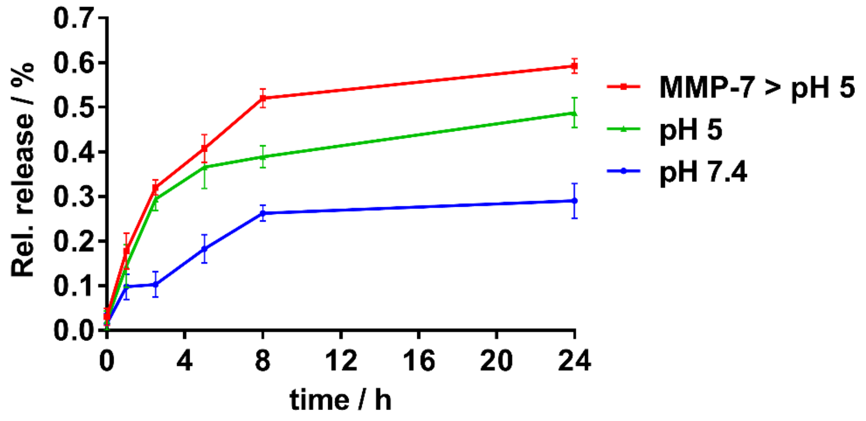

Figure S8. Release of DOX from multistage pNGs under different conditions. The red squares describe the release at pH 5 after the pNG-Dox were digested with MMP-7 overnight.

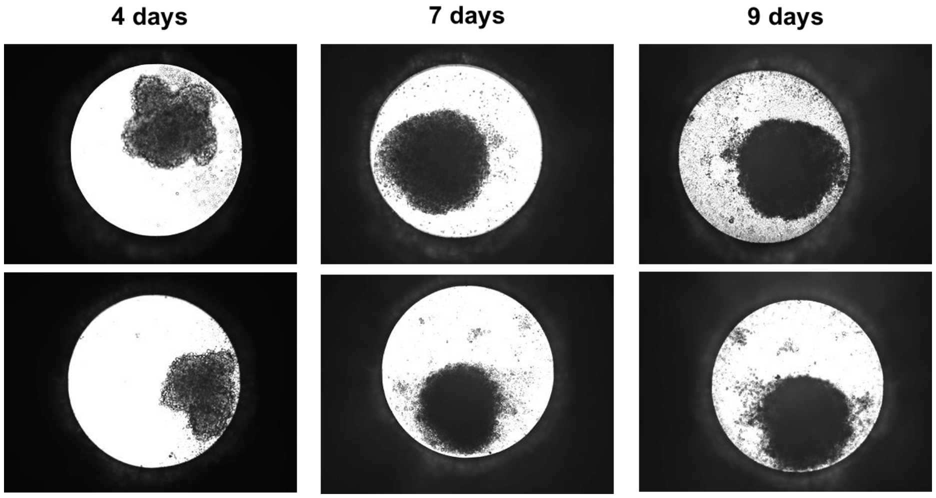

Figure S9. Morphology of multicellular tumor spheroids (MCTS) over time. After 7 days dense, circular spheroids with sizes around 500 µm were obtained.

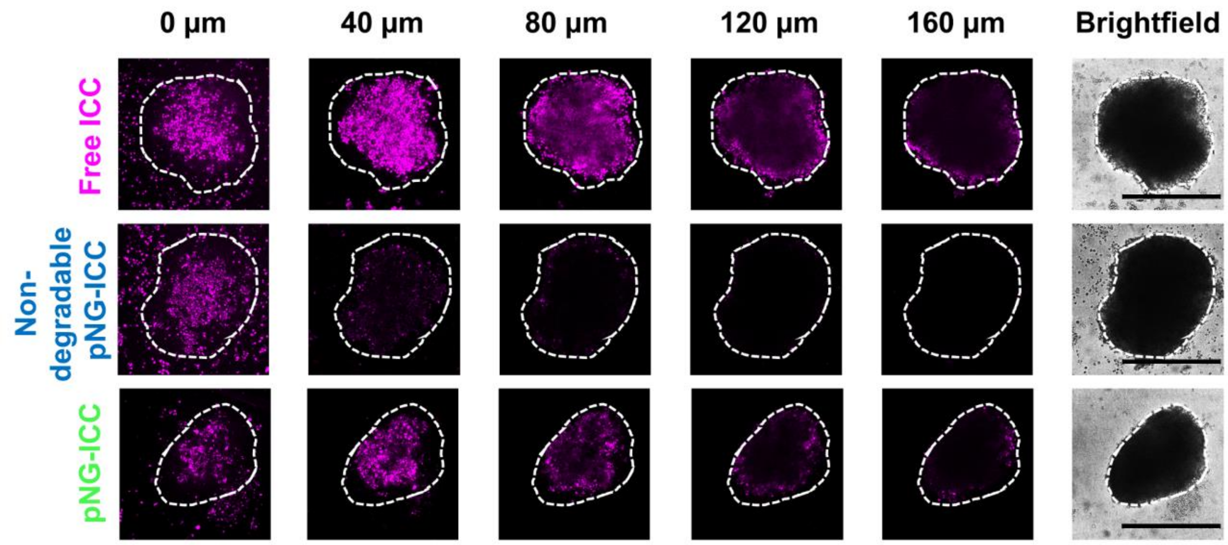

Figure S10. Example of Z-stack optical sections using CLSM of live spheroids incubated for 16 h with free ICC, degradable, or non-degradable pNG-ICC. The white dotted lines display the outside margin of the spheroids in the brightfield image. The black bars in the brightfield images represent 500 µm.

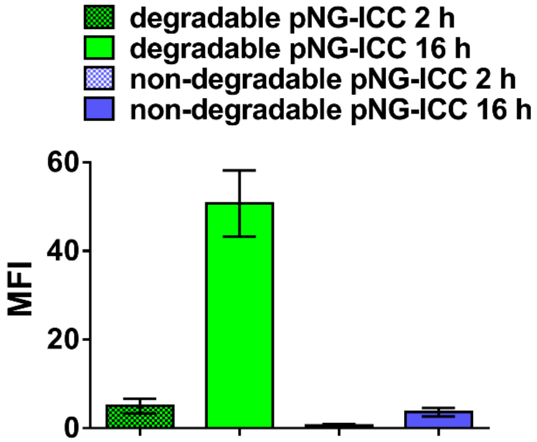

Figure S11. Mean fluorescence intensity over the area of spheroid sections after 2 h and 16 h incubation. Error bars indicate SEM of three spheroid sections per time point and sample.

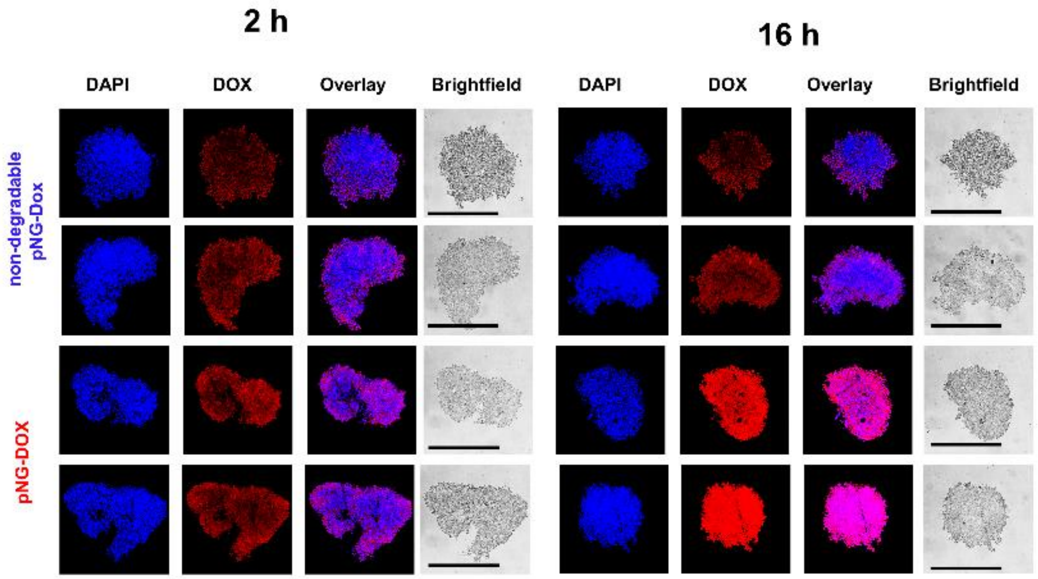

Figure S12. CLSM images of MCTS cryosections with 20-fold magnification. MCTS were incubated with degradable or non-degradable pNG-DOX for a) 2 h and b)16 h, respectively. The black bars in the brightfield images represent 500 µm.

III. **Experimental Data**

(6-maleimidocaproyl) hydrazone derivative of doxorubicin (aldoxorubicin)

^{1}H-NMR (500 MHz, MeOD-d_4, δ): 7.91 (s, 1H), 7.90 (d, *J* = 1.9 Hz, 1H), 7.65 (dd, *J* = 5.7, 4.1 Hz, 1H), 6.98 (s, 2H), 5.77 (t, *J* = 4.8 Hz, 1H), 5.51 (s, 1H), 5.46 (d, *J* = 6.2 Hz, 1H), 5.30 (d, *J* = 2.9 Hz, 1H), 4.95 (t, *J* = 6.7 Hz, 1H), 4.40 (dd, *J* = 7.6, 4.8 Hz, 2H), 4.03 (q, *J* = 6.5, 6.1 Hz, 1H), 3.98 (s, 3H), 3.57 (d, *J* = 4.3 Hz, 1H), 3.25 – 3.20 (m, 2H), 2.75 (d, *J* = 17.3 Hz, 1H), 2.21 (dd, *J* = 15.4, 7.7 Hz, 1H), 2.14 (dd, *J* = 13.4, 6.8 Hz, 1H), 2.07 (s, 2H), 1.89 (td, *J* = 12.6, 3.5 Hz, 1H), 1.73 (dd, *J* = 12.0, 4.1 Hz, 1H), 1.55 – 1.42 (m, 1H), 1.30 (q, *J* = 7.7 Hz, 3H), 1.16 (d, *J* = 6.5 Hz, 3H), 1.02 (p, *J* = 7.9 Hz, 2H) ppm; **HRMS (ESI-TOF) *m*/z**: $[M+H]^+$ calculated for $C37H43N4O13^+$: 751.2821; found: 751.2861; $[M+Na]^+$ calculated for $C37H42N4NaO13^+$: 773.2641; found: 773.2664.

^{1}H-NMR of (6-maleimidocaproyl) hydrazone derivative of doxorubicin (aldoxorubicin)

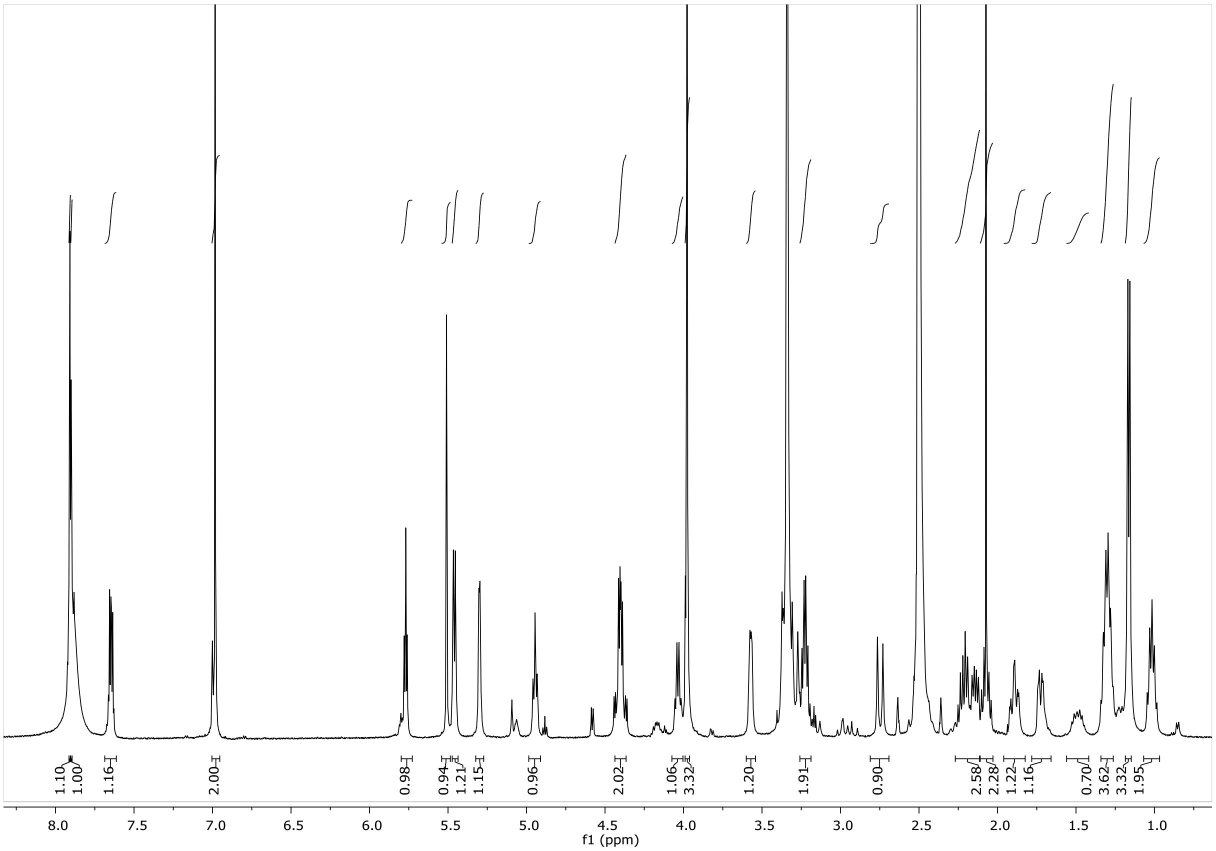

Mass spectrum (ESI-TOF) of (6-maleimidocaproyl) hydrazone derivative of doxorubicin (aldoxorubicin)

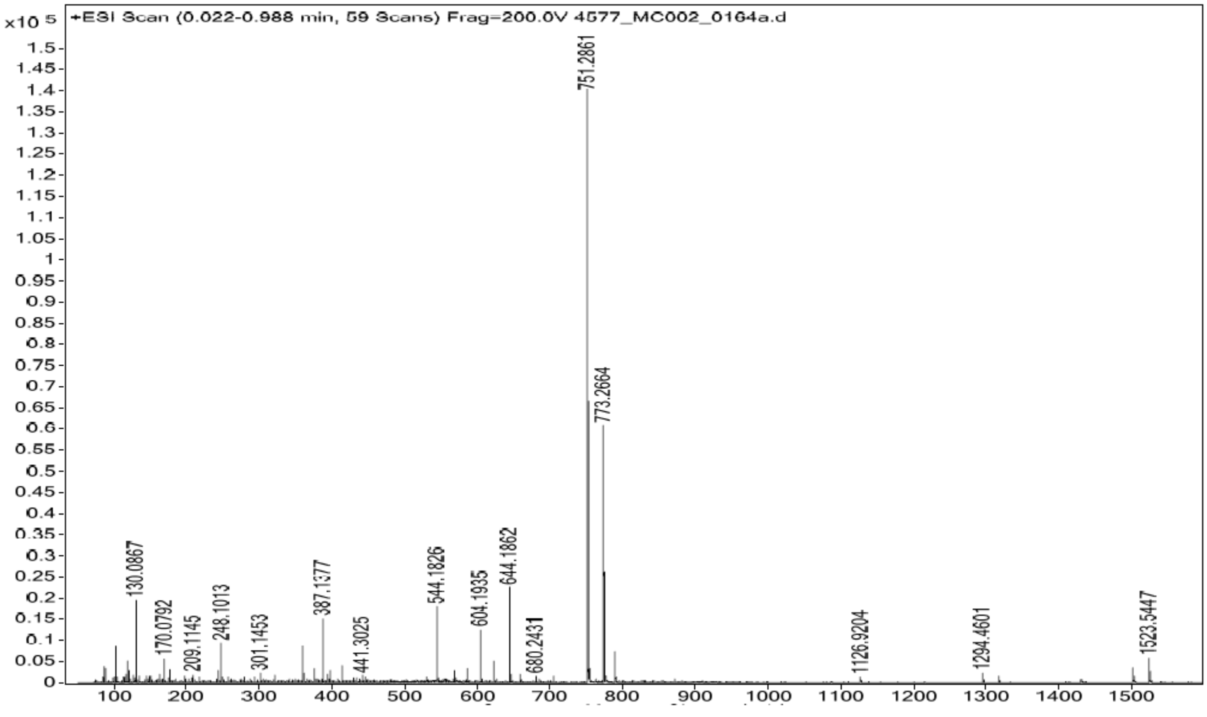

dPG-(1R,8S,9s)-Bicyclo[6.1.0]non-4-yn-9-ylmethyl (dPG-BCN)

^{1}H-NMR (500 MHz, D_2O, δ: 4.24-3.42 (m, 5H, dPG backbone), 2.31-2.14 (m, 6H, cyclooctyne), 1.60-1.28 (m, 2H, cyclooctyne), 0.98-0.80 (m, 3H, cyclopropane) ppm.

4.2% functionalization

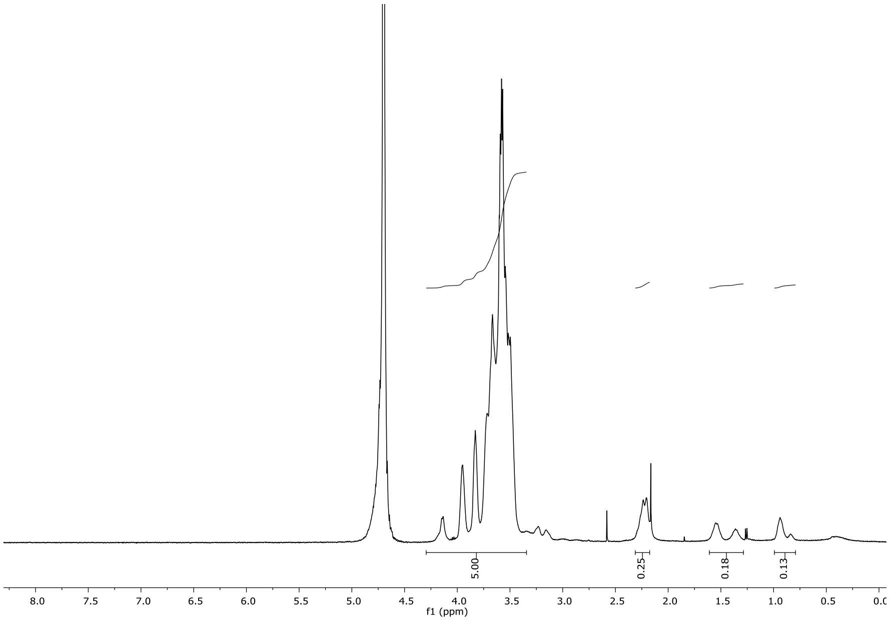

8.0% functionalization

^{1}H-NMR (500 MHz, MeOD-*d*$_4$, δ: 4.24-3.42 (m, 5H, dPG backbone), 2.42-2.50 (m, 6H, cyclooctyne), 1.69-1.28 (m, 2H, cyclooctyne), 1.02-0.80 (m, 3H, cyclopropane) ppm.

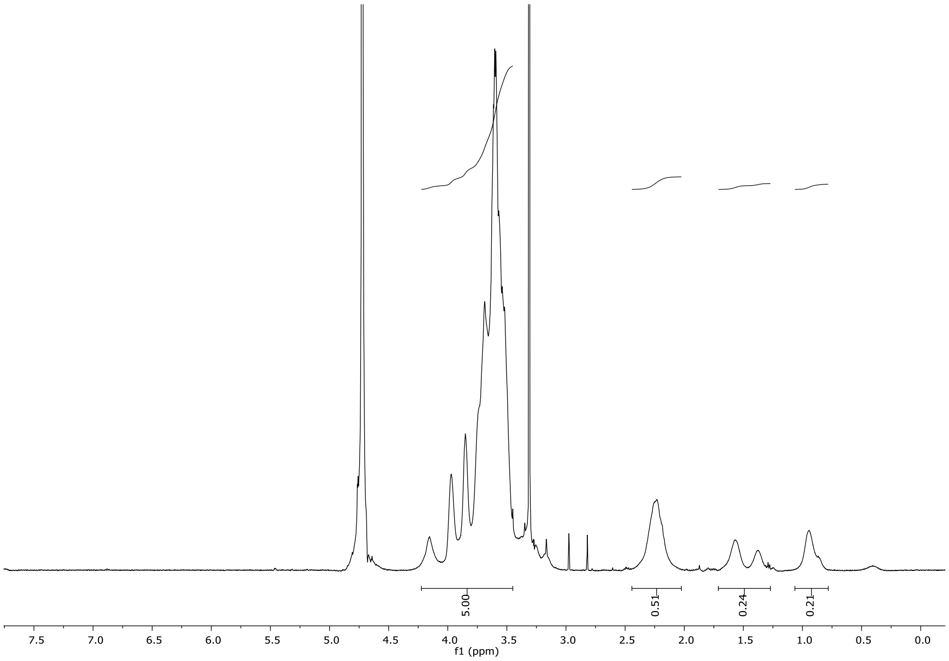

Supplement: Supplementary file 1 — Supplementary information, experimental data, figures, and tables. [file thnov10p0091s1.pdf]
